# A single-cell atlas of glioblastoma evolution under therapy reveals cell-intrinsic and cell-extrinsic therapeutic targets

**DOI:** 10.1038/s43018-022-00475-x

**Published:** 2022-12-20

**Authors:** Lin Wang, Jangham Jung, Husam Babikir, Karin Shamardani, Saket Jain, Xi Feng, Nalin Gupta, Susanna Rosi, Susan Chang, David Raleigh, David Solomon, Joanna J. Phillips, Aaron A. Diaz

**Affiliations:** 1 Department of Neurological Surgery, University of California, San Francisco, San Francisco, CA USA; 2 Department of Pathology, University of California, San Francisco, San Francisco, CA USA

**Keywords:** Tumour heterogeneity, Cancer genomics, Cancer, Cancer therapeutic resistance

## Abstract

Recent longitudinal studies of glioblastoma (GBM) have demonstrated a lack of apparent selection pressure for specific DNA mutations in recurrent disease. Single-cell lineage tracing has shown that GBM cells possess a high degree of plasticity. Together this suggests that phenotype switching, as opposed to genetic evolution, may be the escape mechanism that explains the failure of precision therapies to date. We profiled 86 primary-recurrent patient-matched paired GBM specimens with single-nucleus RNA, single-cell open-chromatin, DNA and spatial transcriptomic/proteomic assays. We found that recurrent GBMs are characterized by a shift to a mesenchymal phenotype. We show that the mesenchymal state is mediated by activator protein 1. Increased T-cell abundance at recurrence was prognostic and correlated with hypermutation status. We identified tumor-supportive networks of paracrine and autocrine signals between GBM cells, nonmalignant neuroglia and immune cells. We present cell-intrinsic and cell-extrinsic targets and a single-cell multiomics atlas of GBM under therapy.

## Abstract (web summary)

Wang et. al. perform single-cell and spatial analyses of paired primary and recurrent samples from patients receiving standard-of-care therapy for GBM and find changes in tumor signaling pathways and the microenvironment with targetable potential.

## Main

The genetics of glioblastoma (GBM), the most common and aggressive primary malignancy of the adult brain, have been extensively characterized^1–3^. Despite this fact, GBMs have proven resistant to all genotoxic therapies employed in clinical trials thus far. Recent longitudinal studies of GBMs based on bulk DNA sequencing demonstrate a lack of selection pressure for DNA mutations that are private to either primary or recurrent disease, a lack of association between genetic selection pressure and standard therapy and a remarkable clonal stability under therapy^4^. These findings are consistent with recent molecular analyses of spatially mapped biopsies from GBM specimens, which find minimal evidence of intra-tumor regional heterogeneity in clonal mutations^5^. Taken together, these results support the hypothesis that selection pressure for or against specific mutations occurs mostly during initial malignant transformation and that standard chemoradiation therapy does not apply significant additional selection pressure at the level of genomic alteration.

**Fig. 1:**
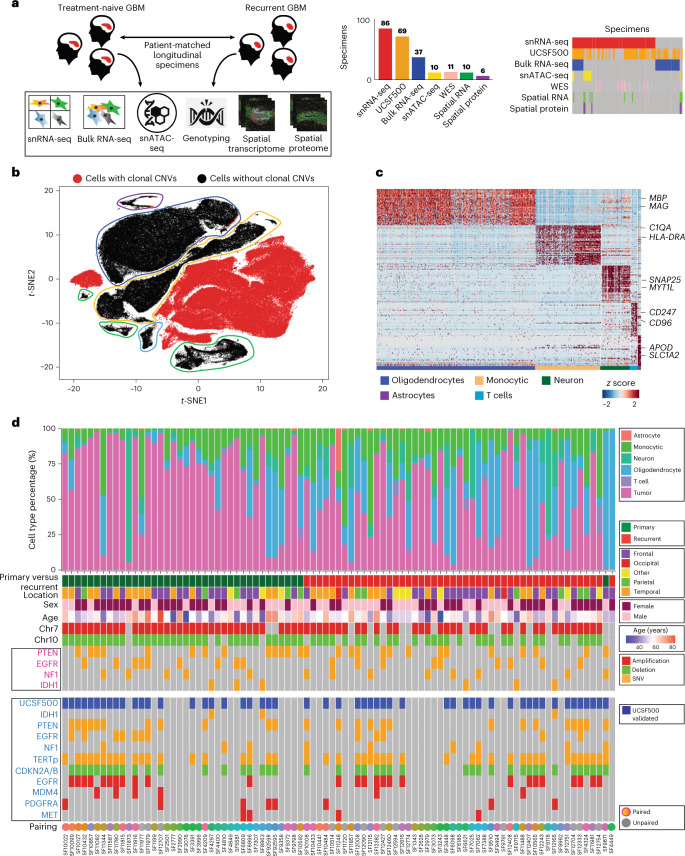
A single-cell RNA atlas of human glioblastoma through recurrence. **a**, An overview of genomics studies on paired longitudinal GBM specimens. **b**, A *t*-distributed stochastic neighbor embedding (*t-*SNE) of the first ten principal components of snRNA-seq data. Cells with CNVs are annotated. *n* = 86 tumors were used (**b**–**d**). **c**, A hierarchical clustering of cells without CNVs, with several cluster-specific genes highlighted. **d**, A summary of sample cellular composition, genotype and demographics. Top: cellular composition inferred from snRNA-seq. Middle: patient and sample annotations, with genotype inferred from snRNA-seq. Bottom: genotypes inferred from the UCSF500 clinical DNA-amplicon-sequencing assay performed on adjacent tissue and controlled by sequencing a patient-matched blood specimen.

On the other hand, recent single-cell/single-nucleus RNA-sequencing (sc/snRNA-seq) studies of primary disease have demonstrated that GBM cells exhibit a high degree of plasticity at the phenotypic level and apparent transitions between cellular states^6–9^; however, it is unknown to what extent standard therapy, temozolomide (TMZ) chemotherapy, ionizing radiation (IR) and surgical resection, applies a selection pressure for or against specific cell types at recurrence. The extent to which standard therapy shapes the milieu of tumor-associated immune cells and nonmalignant neuroglia is not fully understood. To address this, we performed snRNA-seq on a cohort of patient-matched primary-recurrent paired specimens (*n* = 86), together with single-cell assay for transposase-accessible chromatin via sequencing (scATAC-seq), spatial transcriptomics (ST), spatial proteomics (SP) and exome sequencing (exome-seq) for specific subcohorts (Fig. 1a). We present a single-cell multi-omics atlas of GBM under therapy. This resource fills a gap in our knowledge of GBM at recurrence and has allowed us to derive several clinically relevant findings.

We extend our previous finding that proliferating GBM cells lie on a single axis of variation, ranging from the Verhaak proneural (PN) to mesenchymal (MES) phenotypes to the context of recurrent GBM. On average, we found that GBM patients undergo a PN-to-MES shift at recurrence, concomitant with an increase in the birth rate of MES cells in recurrent tumors and supported by paracrine signals from the tumor microenvironment. We identified gene-expression correlates of the re-entry of previously quiescent MES cells into the cell cycle at recurrence and found that targeting these genes decreases GBM cell viability. We identified chromosome-arm-scale copy-number variants (CNVs) that correlate with the MES phenotype, not previously described. Hypermutation status was found to be a predictor of increased T-cell infiltration at recurrence. Moreover, increased T-cell infiltration at recurrence was prognostic. Last, we mapped intercellular paracrine and autocrine signals between neoplastic cells, non-neoplastic neuroglia and immune cells via snRNA-seq. We validated coexpression of these signals in situ via ST and SP. We showed that these signals enhance GBM cell viability in a panel of low-passage patient-derived cell lines and are targetable therapeutically. Taken together, these studies address fundamental questions of GBM cellular biology and the selection pressure applied by standard therapy, as well as provide therapeutic targets for further development.

## Results

### Single-cell transcriptomics of longitudinal GBM specimens

**Extended Data Fig. 1:**
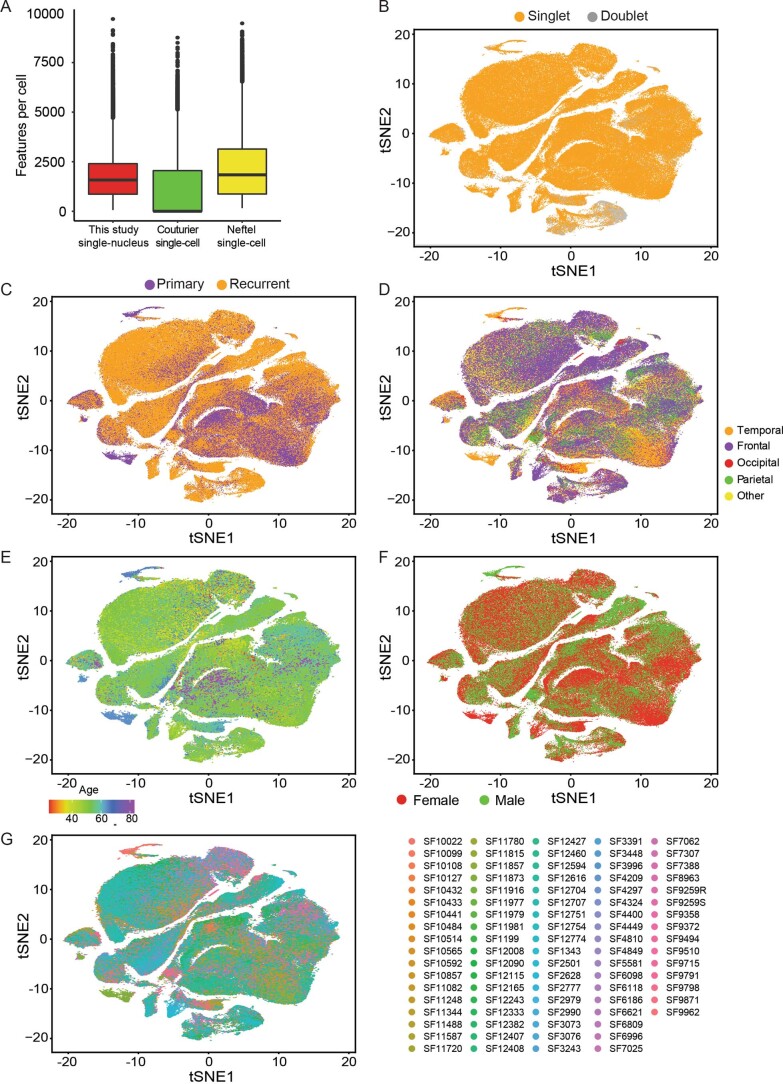
ScRNA-seq preprocessing and QC. **a**) Feature counts per cell compared between the single-nucleus RNA-seq from this study and single-cell data from recent studies of GBM, all data were acquired via the 10X Genomics platform. The numbers biologically independent cells used for this panel were: N = 93,032 (our study), 22,559 (Couturier), and 12,010 (Neftel). Boxplots are defined as follows, lower/upper whiskers: smallest/largest observation ≥/≤ the lower/upper hinge −/+ 1.5 times the interquartile range (IQR); lower/upper hinge: 25^th^/75^th^ percentile; center: 50^th^ percentile. **b**) TSNE plot of the first 10 principal components of the snRNA-seq data with inferred doublet events highlighted. N = 86 tumors shown in panels B-G. **c)-g**), as in **b**), but primary-vs-recurrent, location of specimen resection, patient age, sex, and identifier annotated respectively.

We profiled 86 longitudinal fresh-frozen tissue specimens from 49 patients undergoing surgical resection for GBM via snRNA-seq. For 36 patients we profiled paired specimens from the primary untreated tumor and matched first recurrence (Fig. 1a and Supplementary Table 1), additionally we profiled four untreated-primary and six first recurrence unmatched specimens. At the time of the first recurrence all patients had been treated only with standard-of-care therapy (TMZ, IR and surgical resection). The cohort’s age ranged from 35–76 years and had a male-to-female ratio of 1.2. Nuclei isolation from frozen tissue, nuclei capture and library preparation were performed as previously described^8^, yielding 254,288 transcriptomes. We found that data quality metrics for our single-nucleus data, such as number of features per cell (Extended Data Fig. 1a) or doublet rate (Extended Data Fig. 1b), met or exceeded the quality for recent GBM single-cell studies^7,9^. Neoplastic cells (daughters of the tumor-initiating cell) were separated from nonmalignant neuroglia, endothelial and immune cells via our previously described approaches^10–12^, which include an analysis of gene signatures as well as expressed mutations (Fig. 1b–d). Mutation profiles were validated by DNA-amplicon sequencing via the UCSF500 panel for 52 of 86 cases, the mutational status of isocitrate dehydrogenase (IDH) was assessed for all patients via sequencing and and/or immunohistochemistry. Four samples were identified as having mutations in IDH1 and were excluded from further analysis. We use the term GBM to refer to IDH-wild-type GBM from hereafter. On average, samples from recurrent tumors had less purity than primary cases (Fig. 1d and Extended Data Fig. 1c). All cell types found were represented in specimens from across all lobes of the brain, a spectrum of ages and both sexes (Extended Data Fig. 1d–g).

### Meta-analysis supports a proneural–mesenchymal axis

**Fig. 2:**
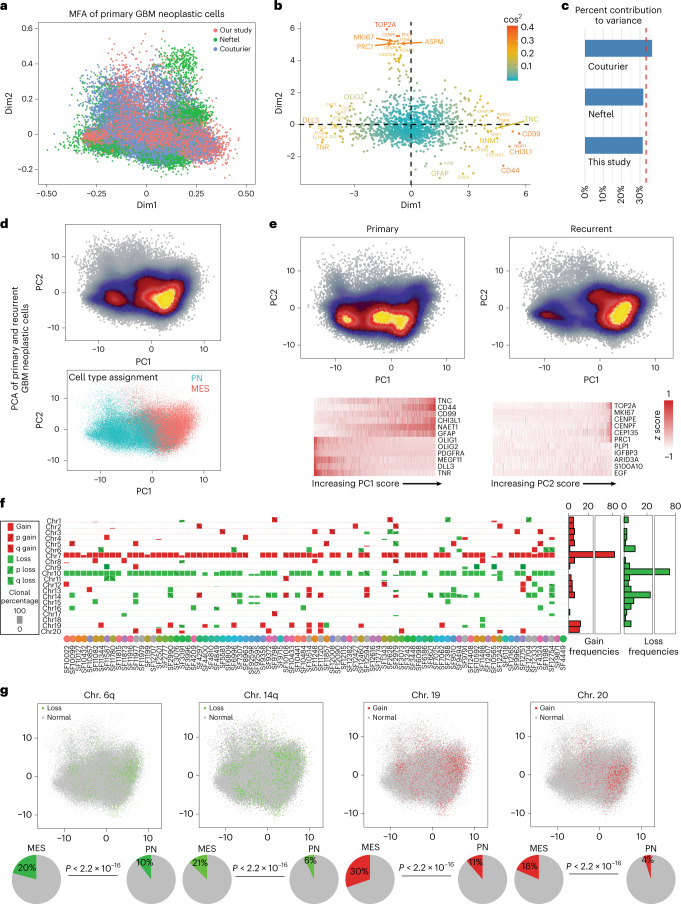
A meta-analysis of public and in-house data identifies the proneural-to-mesenchymal axis as the primary source of phenotypic variation in glioblastoma neoplastic cells and genetic correlates. **a**–**c**, MFA of primary GBM neoplastic cells from the scRNA-seq data of Neftel et al.^9^ (*n* = 5,588 cells), Couturier et al.^7^ (*n* = 17,884 cells) and snRNA-seq from our study (*n* = 34,582 cells). Cell loadings (**a**), gene scores (**b**) and an analysis of each dataset’s contribution to variance explained (**c**). **d**, Top: PCA of all GBM neoplastic cells from our study from longitudinal specimens. *n* = 78,415 cells from 62 paired tumors. Bottom: PN and MES cell-type assignments. **e**, Separate plots of the cells from primary GBMs (left) and recurrent cases (right). Expression values of top-loading genes in single cells are shown below. Cells are sorted according to position along the axis labeled. *n* = 78,415 cells. **f**, Summary of megabase-scale CNVs detected in the snRNA-seq data, indicating the presence of CNVs in individual samples, their type and cellular frequency. *n* = 86 tumors. **g**, The distribution of Chr6^−^, Chr14^−^, Chr19^+^ and Chr20^+^ CNVs in single cells in PCA from **d**. Bottom: percentages of PN and MES cells that have these genotypes and the associated one-sided Fisher’s *P* value indicating the probability that this association occurs by chance. *n* = 78,415 cells.

**Extended Data Fig. 2:**
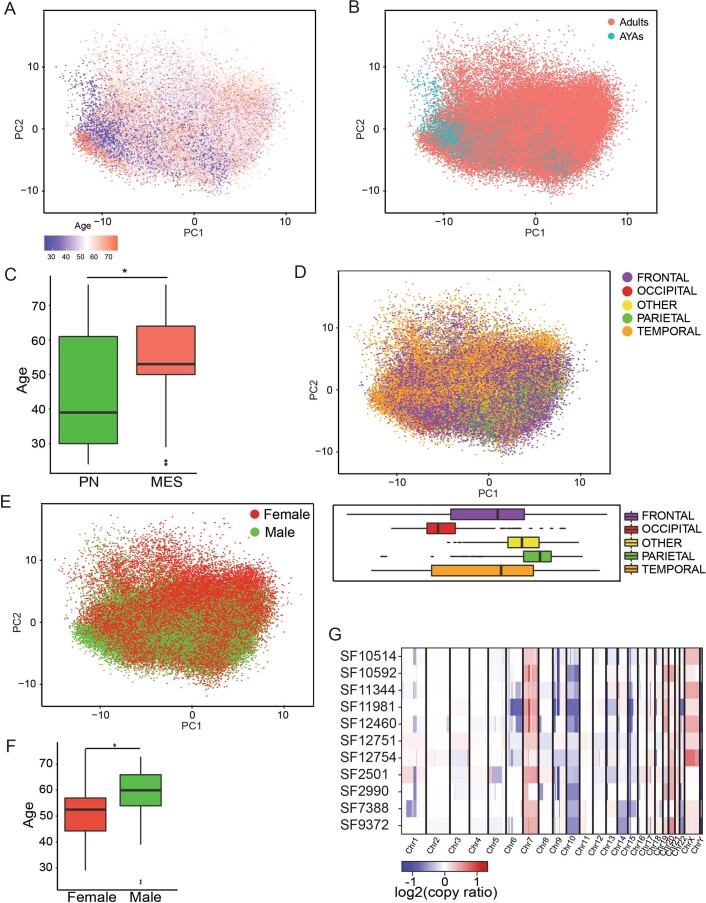
Clinical and genomic correlates of expression. **a**) A PCA of GBM neoplastic cells from snRNA-seq with patient age annotated, N = 78,415 cells. **b**) Annotation of adult vs. adolescent and young-adult status, N = 78,415 cells. **c**) A comparison of patient age between snRNA-seq neoplastic cells classified as PN vs. MES, N = 72 tumors. *- one-sided Wilcoxon rank-sum test p = 0.0161. Boxplots in panels C and E-F are defined as follows, lower/upper whiskers: smallest/largest observation ≥/≤ the lower/upper hinge −/+ 1.5 times the interquartile range (IQR); lower/upper hinge: 25^th^/75^th^ percentile; center: 50^th^ percentile. **d**) A PCA of GBM neoplastic cells from snRNA-seq with location of resection annotated. The distributions along principal component one of cells by location are shown as boxplots below, N = 78,415 cells. **e**) As above, but with sex annotated, N = 72 tumors. **f**) A comparison of patient ages between the male and female sex, N = 72 tumors. *- one-sided Wilcoxon rank-sum test p = 0.0389. **g**) CNV calls from exome-seq for select specimens.

We recently showed that the phenotypes of proliferating primary GBM cells have a dominant axis of variation that ranges from the MES to PN transcriptional subtypes^8^. Subsequent scRNA-seq studies of primary GBM specimens and patient-derived tumor-propagating cells have produced similar findings^6^. We performed a meta-analysis of snRNA-seq data from our primary tumors and other recent single-cell studies of primary GBMs^7,9^. An unbiased multiple-factor analysis (MFA; Methods), an extension of principal-component analysis (PCA) to multiple tables, showed that the largest contribution to variation in primary GBM neoplastic cells was an axis between MES (for example *CD44* and *CHI3L1*) and PN (for example *OLIG2* and *DLL3*) expression programs (Fig. 2a,b and Supplementary Tables 2 and 3). Inter-table analysis demonstrated nearly equal contribution to overall variance from each of the studies included, indicating that this result was not due to inter-laboratory technical effects (Fig. 2c). The second largest source of variation in this analysis, consistent with Wang et al., was the expression of markers of mitotic cells such as *MKI67*. We found that the same result held in both primary and recurrent specimens; however, the distribution of cells along the PN–MES axis shifts at recurrence (Fig. 2d,e). Using our previously described approach, we classified all neoplastic cells as either PN or MES^11^. This classification agreed with PCA analysis (Fig. 2d,e). We found a significant association between patient age and the MES phenotype (Extended Data Fig. 2a–c); however, there was no such association with tumor location (Extended Data Fig. 2d). While we did see an association between age and sex in our data, we did not find a significant association between sex and the MES phenotype (Extended Data Fig. 2e,f). Last, we identified megabase-scale CNVs in the snRNA-seq data using patient-matched exome sequencing (exome-seq) as validation (Extended Data Fig. 2g and Methods). We found significant associations with between several prevalent chromosome-arm level CNVs and the MES phenotype (Fig. 2f,g and Extended Data Fig. 2g).

### A mesenchymal shift in recurrent disease

**Fig. 3:**
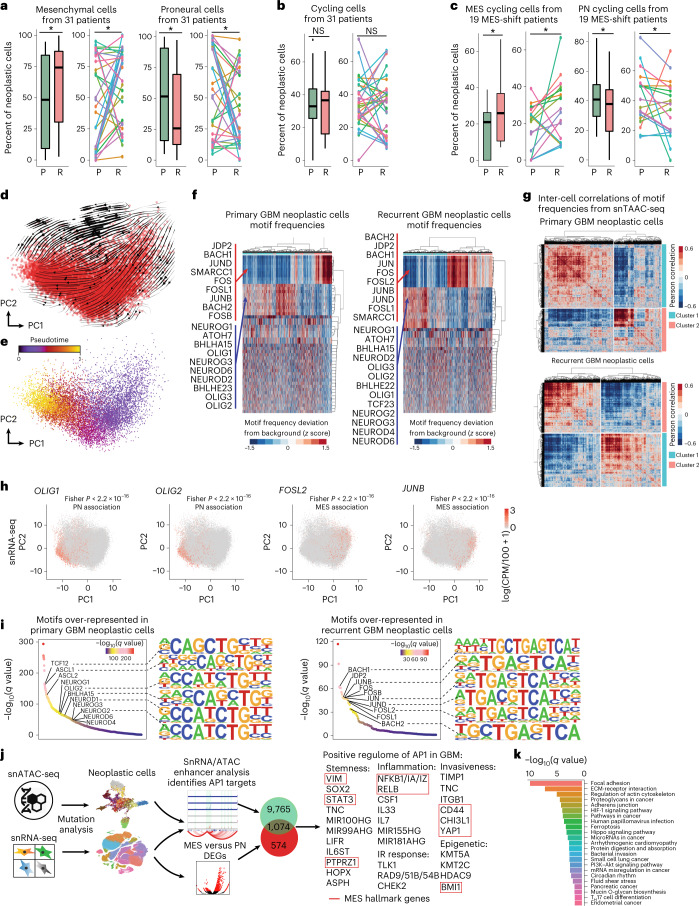
A proneural-to-mesenchymal shift is observed in GBM at recurrence, driven by an increase in cycling mesenchymal cells and mediated by AP1. **a**, Percentages of PN and MES neoplastic cells in patient-matched paired primary and recurrent specimens via snRNA-seq (*P* = 0.03967). **b**, The percentages of cycling neoplastic cells in primary and recurrent samples. **c**, The percentages of PN and MES cycling cells for paired cases undergoing PN-to-MES shift (*P* = 0.01565). Paired longitudinal samples were used (**a**–**c**); *n* = 62 paired samples from 31 patients (**a**,**b**) and *n* = 38 samples from 19 patients who underwent MES transition (**c**). Boxplot lower/upper whiskers indicate the smallest/largest observation ≥/≤ the lower/upper hinge ± 1.5 times the interquartile range (IQR); lower/upper hinge indicates 25th/75th percentiles; and the center indicates 50th percentile. A one-sided Wilcoxon signed-rank test for paired samples was used. *P 0.05. P, primary; R, recurrent. **d**, RNA velocities and associated field lines for *n* = 10,456 MES cells from recurrent GBMs, visualized via PCA. **e**, Inference of pseudotime based on the flow field in **d**. *n* = 10,456 cells. **f**, Heat maps comparing cell-by-motif matrices of transcription factor motif deviances between primary and recurrent GBMs, derived from snATAC-seq of *n* = 3,894 neoplastic cells from primary tumors and *n* = 7,087 neoplastic cells from recurrent tumors. **g**, Heat maps of snATAC-seq inter-cell correlations of transcription factor motif frequencies obtained as deviances from a data-driven background distribution, compared between primary and recurrent neoplastic cells. *n* = 3,894 primary GBM and *n* = 7,087 recurrent GBM derived cells. **h**, Scatter-plots of proneural and AP1 transcription factor expression in snRNA-seq from *n* = 78,415 neoplastic cells show significant (one-sided Fisher’s *P* < 2 × 10^−16^) association with PN and MES cells, respectively. **i**, Over-represented (*q* < 0.05) transcription factor motifs in snATAC-seq reads from *n* = 3,894 primary (left) and *n* = 7,087 recurrent (right) neoplastic cells. Significance was assessed with a two-sided *t*-test and adjusted for multiple hypothesis testing via Storey’s method. **j**, A summary of the AP1 regulome, consisting of genes that are both upregulated in MES cells in the snRNA-seq data and also show correlated enhancer activity at nearby AP1 binding sites, specifically in MES cells from the snATAC-seq data. **k**, KEGG pathway analysis of the inferred AP1 regulome.

**Extended Data Fig. 3:**
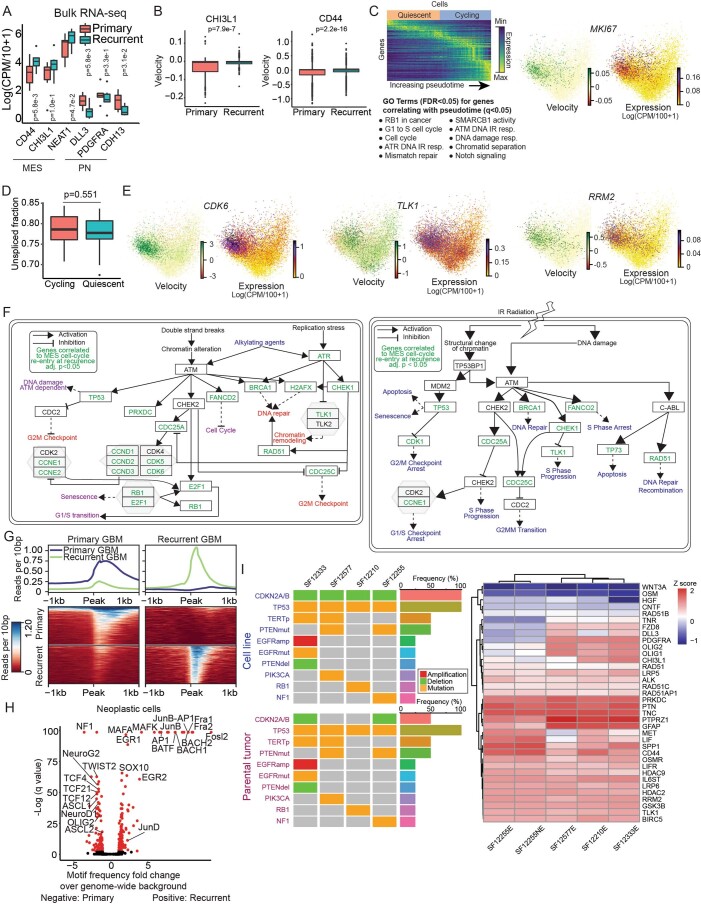
Validation, correlates, and models of the MES shift. **a**) Comparison of PN, MES and monocytic-lineage cell marker genes between primary and recurrent bulk RNA-seq of patient-matched GBM longitudinal specimens, N = 30 tumors. A one-sided paired T-test was used to assess significance. Boxplots in panels A-B and D are defined as follows, lower/upper whiskers: smallest/largest observation ≥/≤ the lower/upper hinge −/+ 1.5 times the interquartile range (IQR); lower/upper hinge: 25^th^/75^th^ percentile; center: 50^th^ percentile. **b**) Boxplots of RNA velocities for MES hallmark genes *CD44*, *CHI3L1* computed over PN neoplastic cells from recurrent GBM cases, N = 37,428 cells. A one-sided signed Wilcoxon rank-sum test was used to assess significance. **c**) (Left) A heatmap representation of the pseudotime inference shown in Fig. 3d, e is shown above. Gene ontology terms from WikiPathway.org that are over-represented (FDR < 0.05) based on genes that correlate with pseudotime, is shown below. (Right) RNA velocity and expression for *MKI67* in MES neoplastic cells from recurrent GBMs, visualized in PCA space. **d**) The fractions of total spliced and unspliced mRNAs, compared between cycling and quiescent MES cells from recurrent cases using a two-sided Wilcoxon rank-sum test, p = 0.0551, N = 10,456 cells. **e**) RNA velocity and expression for *CDK6*, *TLK1*, and *RRM2* in MES neoplastic cells from recurrent GBMs, visualized in PCA space, N = 10,456 cells. **f**) (Left) The DNA-damage response pathway adapted from Wikipathway.org. (Right) The response to IR pathway adapted from Wikipathway.org. Genes correlating with cell-cycle re-entry by MES cells at recurrence are annotated in green. **g**) Read-density and heatmap plots summarizing reads mapping to primary- and recurrent-specific scATAC-seq peaks respectively, from N = 10,981 cells. **h**) Over-represented transcription factor motifs in primary- and recurrent-specific scATAC-seq peaks, from N = 10,981 cells. **i**) (left) A comparison of genotypes between each of the patient-derived cell lines and the tumor specimens from which they were derived, performed via UCSF500 clinical genotyping. (right) Expression levels via RNA-seq of the patient-derived cell lines for PN and MES markers and genes targeted in in vitro assays.

A shift toward the MES phenotype at recurrence is a hallmark of therapy resistance^3,13–15^. Considering paired cases only and using a paired test, we found a significant increase in the percentage of MES cells per patient on average at recurrence (Fig. 3a). This MES shift was also observed in our bulk RNA-seq data (Extended Data Fig. 3a). While we found no significant difference in the percentage of cycling cells overall when comparing paired primary and recurrent cases (Fig. 3b), cases that underwent a MES shift showed a marked increase in the percentage of MES cycling cells at recurrence (Fig. 3c). The MES shift, as previously described^3^, could in principle be explained by at least several factors: a preferential resistance of MES cells to therapy, activation of a MES expression program within non-MES cells and/or a change in the birth rates of non-MES and MES cells at recurrence. We found that the latter, an increase in the proliferation rate within the MES population, has a clear contribution. When we performed RNA velocity analysis to estimate rates of gene transcription (Methods) we found modest and infrequent positive velocities for MES genes, in non-MES cells (Extended Data Fig. 3b). PCA of MES cells from recurrent cases identified cycling versus quiescence as their primary axis of variation. RNA velocities indicated a unidirectional transition from quiescent to cycling MES cells (Fig. 3d,e and Extended Data Fig. 3c). There was no significant difference in the fractions of unspliced versus spliced total transcripts observed when comparing cycling to quiescent populations (Extended Data Fig. 3d), indicating that this result is not due to technical bias. Genes that correlated with progression from quiescence to cycling (Supplementary Table 4) were over-represented in the DNA-damage response pathway (Extended Data Fig. 3c,e,f). Conversely, transforming growth factor-β pathway genes were upregulated in quiescent MES cells.

### Single-cell open-chromatin profiling implicates AP1

We performed scATAC-seq on 10 IDH-wild-type GBMs: four primary-recurrent pairs, one unmatched primary case and one unmatched recurrent case. This yielded 22,214 sequenced cells. Additionally, we included in our analysis four primary IDH-wild-type GBMs that we had profiled via snATAC-seq previously^8^. Neoplastic cells were separated from immune cells and nonmalignant glia based on detected mutations and a clustering of gene activity profiles. To identify cell states based on transcription factor activity, we scanned scATAC-seq reads for over-represented transcription factor motifs compared to a data-driven background model (Methods). Neoplastic cells were then hierarchically clustered based on inter-cell correlations in motif frequency deviances from background, identifying clusters of cells with similar transcription factor utilization (Fig. 3f,g). In both the primary and recurrent GBM hierarchical clusters, at their top levels, neoplastic cells were split into two states that bore hallmarks of the PN and MES phenotypes, respectively. In particular, the first cluster was enriched for proneural transcription factors (for example OLIG2 and NEUROG1). The second had activator protein 1 (AP1) complex components over-represented, some of which have been previously described as regulating MES gene expression^16^. Motif analysis of scATAC-seq provides information about differential targeting of transcription factors. This is often independent of differential transcription factor expression per se. Nonetheless, we did find that some of these cluster-specific transcription factors were also differentially expressed between PN and MES cells in our snRNA-seq data (Fig. 3h), further supporting our interpretation of these two clusters as consisting of PN and MES cells, respectively. Consistent with the MES shift identified in our snRNA-seq data, we also found evidence for the MES shift in our scATAC-seq data. In particular, the MES cluster increased in relative size at recurrence (primary, 60% PN and 40% MES; recurrent, 51% PN and 49% MES; Fig. 3f,g). Moreover, the PN and MES cells became more polarized in their respective phenotypes at recurrence with the MES cluster becoming more homogenous; median intra-MES cell correlation increased from 0.08 to 0.20, whereas PN-to-MES cell correlations dropped from −0.12 to −0.180.18 in the median. A differential motif-enrichment test confirmed that PN and MES transcription factors were over-represented in primary and recurrent GBM specimens, respectively (Fig. 3i and Supplementary Table 5). Last, when we identified peaks from the snATAC-seq data we found the same enrichments for proneural and AP1 transcription factor motifs in the peaks specific to primary versus recurrent cases, respectively (Extended Data Fig. 3g,h and Supplementary Tables 6–9).

### Radiation induced AP1 enhances mesenchymal genes

**Fig. 4:**
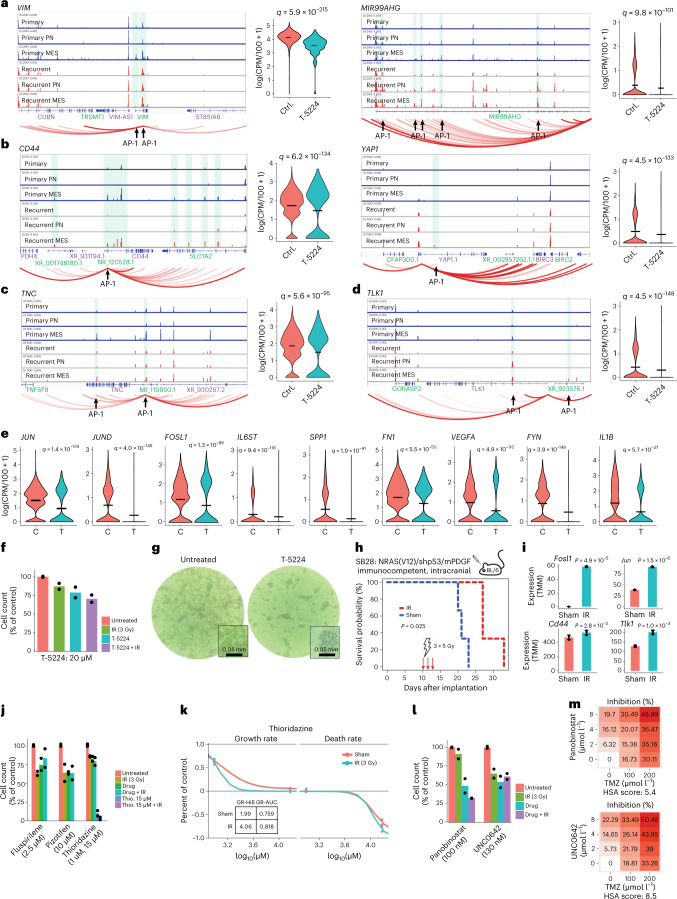
AP1 positively regulates the mesenchymal phenotype and is induced by ionizing radiation. **a**–**d**, Left: enhancer activity analysis identifies enhancers that correlate with nearby gene expression, contain AP1 recognition motifs and are differentially active in human MES versus PN cells, from *n* = 20,544 cells. Right: concomitant decreases in gene expression after AP1 inhibition, observed in low-passage GBM cultures treated for 48 h with T-5224. *n* = 3,593 cells. **e**, Other significant changes in gene expression following AP1-inhibitor treatment. *n* = 3,593 cells. Significance was assessed with a two-sided likelihood-ratio test between hurdle models and adjusted for multiple hypothesis testing via the Benjamini–Hochberg method (**a**–**e**). **f**, Cell proliferation following a 3-d AP1 inhibitor treatment and 48 h after treatment with 3 Gy of IR, for *n* = 2 independent experiments. **g**, Images of AP1 inhibitor-treated and control cultures, representative of *n* = 3 independent experiments. Under AP1 inhibition, monolayer-cultured GBM cells (left) detach from the BME-coated plate and continue to grow as floating spheroids. **h**, Survival for IR-treated (days 10, 12, 14 at 3 Gy d^−1^) cases and controls. Immunocompetent mice were injected intracranially with syngeneic glioma cells (SB28). *n* = 3 mice per condition. Significance is assessed with a log-rank test. **i**, Differences in AP1 and MES-signature gene expression in IR-treated versus control mice, for *n* = 3 mice per condition. Data are presented as mean ± s.d. Significance is assessed via a one-sided *t*-test. **j**, Cell proliferation under combination treatment of IR and BBB-penetrant antipsychotics with inferred off-label activity against TLK1, for *n* = 2 independent experiments. **k**, Inhibition and death rates for the antipsychotic thioridazine, with and without IR, from *n* = 2 independent experiments, indicating synergy between thioridazine and IR. **l**,**m**, Cell proliferation following treatment with an HDAC inhibitor (panobinostat) or a methyltransferase inhibitor (UNC0642), in combination with IR or TMZ. *n* = 2 independent experiments (**l**). Synergy is assessed via highest single agent (HAS) score from *n* = 3 independent experiments (**m**). Source data

**Extended Data Fig. 4:**
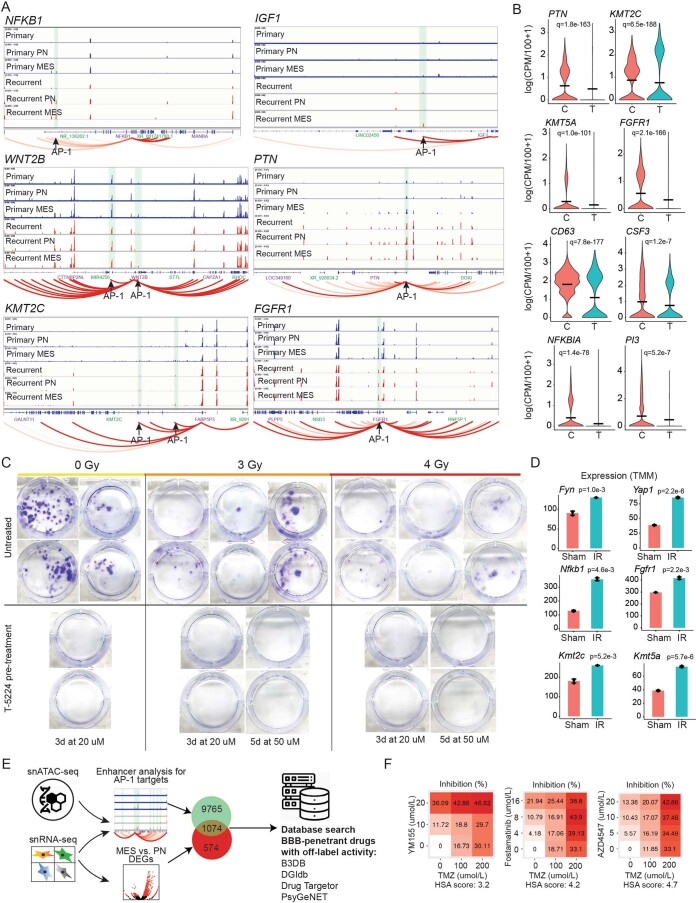
Enhancer, in vitro, and in vivo analysis of the AP1 regulome. **a**) Enhancer activity analysis, as in Fig. 4a. **b**) Gene expression differences between AP1 inhibitor-treated and control GBM cells following 48 hr AP1-inhibitor treatment at 20uM; C: control, T: treatment, *- q < 0.05, where q is the Benjamini-Hochberg adjusted p-value from the MAST two-sided likelihood-ratio test. N = 3,593 cells. **c**) A colony-formation assay with IR treatment and AP1-inhibitor pre-treatment shows that AP1 inhibition abrogates colony formation in BME **d**) Differential gene expression between IR-treated and control murine immunocompetent intracranial gliomas. N = 3 mice per condition were used. Data are presented as mean values + /- standard deviation. A one-sided T-test was used to assess significance. **e**) An overview of the pipeline employed to identify BBB-penetrant drugs that could be repurposed to blockade AP1 targets. **f**) Cell viability post treatment with the indicated agent, in combination with TMZ. Listed beneath is the HSA synergy score for each combination, N = 3 independent experiments. Source data

We sought to elucidate the AP1 regulome in GBM and determine whether it could be targeted for therapeutic benefit. To accomplish this, we correlated snRNA-seq gene expression with scATAC-seq chromatin accessibility in *cis*, using a latent-space approach to infer *cis*-regulatory enhancer activity (Methods). We then scanned these enhancers for AP1 recognition motifs and cross-referenced the results with genes that were differentially expressed between PN and MES neoplastic cells. This identified genes specifically expressed in MES cells, with MES-specific *cis*-regulatory enhancers that are targeted by AP1 (Fig. 3j,k). All previously described hallmarks of the MES phenotype^16–18^ were thusly identified as part of the AP1 regulome in GBM. To functionally validate AP1 regulation of these genes, we exploited low-passage (p3–6) cell lines derived from specimens of human recurrent GBMs, cultured as monolayers in defined factors (Methods). We genotyped these lines via UCSF500 DNA-amplicon sequencing and verified that they closely match their parental tumors (Extended Data Fig. 3i). We then treated these cells with an AP1 inhibitor for 48 h and performed scRNA-seq on treated cells and untreated controls (Methods). We found that AP1 inhibition regressed the expression of genes with AP1-regulated enhancers, including genes associated with stemness (for example *VIM* and *MIR99AHG*), MES hallmarks (for example *CD44* and *YAP1*), invasiveness (for example *TNC* and *FN1*), inflammation (for example *NFKB1*, *FYN* and *IL1B*), IR resistance (for example *TLK1*) and others (Fig. 4a–e and Extended Data Fig. 4a,b). All these genes were part of the AP1 regulome inferred from in vivo human data and had MES-specific *cis*-regulatory enhancers that were targeted by AP1. AP1 inhibition did not significantly decrease GBM cell proliferation, although AP1 inhibition did synergize with IR (Fig. 4f). After approximately 48 h of AP1 inhibitor treatment, GBM cells that had been growing as a monolayer on basement membrane extract (BME)-coated plates detached from the plate and continued to grow as floating spheroids (Fig. 4g). Moreover, AP1 inhibition completely abrogated the ability of GBM cells to form colonies in BME. These findings are consistent with our inference of AP1 regulation of TNC, FN1 and other matricellular genes. To determine whether AP1 is induced by IR, we employed an immunocompetent, intracranial murine model^19^. We treated tumor-bearing mice with IR using a fractionated schedule of 5 Gy on days 10, 12 and 14 after implantation (Methods). This resulted in a significant (28%, *P* = 0.039) extension of survival (Fig. 4h). Upon euthanasia, mice were perfused and tissue from the injection site was collected. We observed a significant increase in the expression of AP1-component genes, as well as MES hallmark genes, AP1-regulated mediators of inflammation and the DNA-damage response (Fig. 4i and Extended Data Fig. 4d). Taken together, these results indicate that AP1 is induced in GBM upon IR treatment and that AP1 positively regulates the MES hallmarks of inflammation, IR resistance and invasiveness through *cis*-regulatory enhancers.

### Targeting the AP1 regulome for therapeutic benefit

We screened genes from the AP1 regulome against databases of known drug interactions, including off-label activity (Fig. 4j–m and Extended Data Fig. 4e.f). Drug candidates were further prioritized based on evidence of blood–brain barrier permeability. We identified two antipsychotics and an antidepressant that target the AP1-regulated gene TLK1 (Fig. 4d,i) that showed cytotoxic activity in vitro (Fig. 4j). In particular, thioridazine synergized with IR (Fig. 4k), consistent with the role of TLK1 in DNA-damage repair. Additionally, the histone deacetylase HDAC9 and lysine methyltransferases KMT5A, KMT2C were implicated targets of AP1 (Fig. 3j and Extended Data Fig. 4a,b,d) and inhibiting these pathways showed synergy with IR and TMZ (Fig. 4l,m). Having determined that standard therapy selects for a MES phenotype in neoplastic cells, we next sought to analyze how immune cells in the GBM microenvironment respond to standard of care.

### Tumor-associated innate immune cells show limited activation

**Fig. 5:**
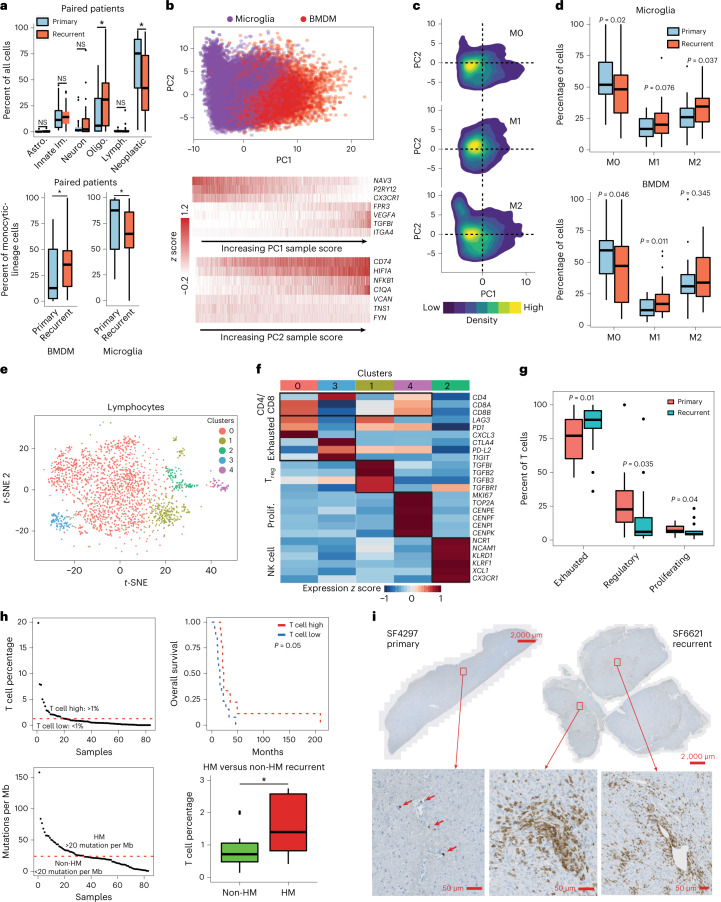
The immune response to standard therapy. **a**, Top: percentages of cell types in primary and recurrent tumors (*n* = 62 tumors). Oligodendrocytes, *P* = 0.0179; neoplastic, *P* = 0.00435; BMDMs, *P* = 0.00862; microglia, *P* = 0.00861. Bottom: percentages of BMDMs/microglia compared between primary and recurrent tumors. **b**, Top: monocytic-lineage cell PCA from *n* = 62 tumors. Bottom: expression levels of top-loading genes for PC1 and PC2 in single cells sorted by sample score. **c**, Distributions of M0, M1 and M2 activation phenotypes in PCA space. **d**, Distributions of innate immune-cell activation phenotypes compared between primary and recurrent specimens. *n* = 62 tumors. Significance assessed via a one-sided *t*-test. **e**, *t-*SNE plot of the first ten PC scores of tumor-associated lymphocytes that have been clustered via Seurat. **f**, Heat map of gene expression in lymphocytes for select cluster-specific genes classifies T cells into proliferative, exhausted and regulatory phenotypes and separates natural killer cells. **g**, Percentages of exhausted, regulatory and proliferating T cells compared between primary and recurrent specimens. A one-sided *t*-test was used to assess significance. *n* = 70 tumors were used (**e**–**g**). **h**, Top left: distribution of T-cell percentages across recurrent samples, with the threshold used to separate relatively T-cell enriched and T-cell poor specimens highlighted. Top right: overall survival, comparing T-cell rich and poor specimens. Significance was assessed via a log-rank test. Bottom left: distribution of mutational burdens across samples, with the threshold used to define hypermutation status highlighted. Bottom right: percentages of T cells compared between hypermutated and non-hypermutated recurrent specimens. Asterisk indicates one-sided Wilcoxon rank-sum test *P* = 0.0493, from *n* = 31 tumors. In boxplots in **a**, **d**, **g** and **h** lower/upper whiskers indicate smallest/largest observation ≥/≤ the lower/upper hinge ± 1.5 × IQR; lower/upper hinge indicates 25th/75th percentile; and center indicates 50th percentile. Patient-matched primary-recurrent paired specimens and the one-sided Wilcoxon signed-rank test for significance were used (**a**,**d**,**g**). *P 0.05. **i**, IHC for CD8 in FFPE specimens, comparing a patient-matched primary and recurrent pair, where the recurrent specimen is an outlier case with T-cell abundance over fourfold greater than average, representative data from four independent experiments with similar results.

Tumor-associated innate immune cells in the aggregate represented 12.6% and 16.5% of cells profiled, on average, in primary and recurrent tumors respectively (Fig. 5a). There was a significant increase in the percent of innate immune cells classified as bone marrow-derived monocytic (BMDM) lineage cells at recurrence and a significant decrease in the relative proportion of central nervous-system-resident microglia. An unbiased PCA of microglia and BMDM cells, including monocytes and their differentiated progeny, identified ontogeny as their primary source of variation (Fig. 5b). The second principal component (PC) stratified cells by the expression of regulators of inflammation (for example *NFKB1*), antigen presentation (for example *CD74*) and reactivity to a hypoxic microenvironment (for example *HIF1A*). We then scored microglia and BMDM for activation status, according to canonical markers of the pro-inflammatory (M1) and inflammation-resolving (M2) phenotypes, against a data-driven background model (Methods). Cells that did not express either program above background levels were classified as M0. We found that M0 cells had negative PC2 scores on average, whereas M1 and M2 cells had positive scores on average (Fig. 5c). This indicates that the second largest source of variation in innate immune cells is activation status. Consistent with recent findings^20^, the majority of GBM-associated innate immune cells resided in an M0 state in primary GBM, based on canonical markers. While there were significant increases in the percentages of activated cells at recurrence, pluralities of both microglia and BMDM remained M0 (Fig. 5d).

### T cells invade the tumor in outliers with superior prognosis

**Extended Data Fig. 5:**
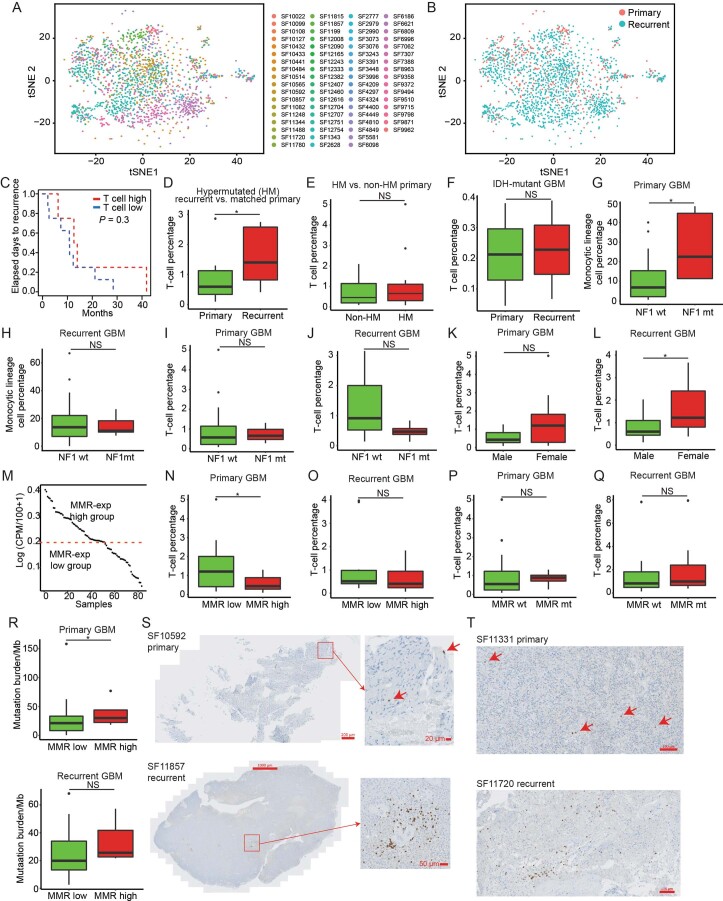
Genetic and clinical correlates of immune phenotypes. The Wilcoxon signed-rank test was used for paired samples and the Wilcoxon rank-sum test was used for unpaired samples for all panels, * - p < 0.05. **a**) A TSNE plot of the first 10 principal components from snRNA-seq of N = 2,247 T cells. **b**) As in **a**), but with primary vs. recurrent annotated, N = 2,247 cells. **c**) Kaplan-Meier analysis of time to recurrence comparing T-cell enriched/poor cases at recurrence. **d**) T-cell percentages in hypermutated (HM) recurrent cases and their matched primary tumors, p = 0.0372. **e**) HM vs. non-HM primary cases show no significant difference in T-cells. **f**) Primary vs. recurrent IDH-mutant GBM T-cell percentages. **g**) NF1-mutant vs. NF1-wildtype primary GBM monocytic lineage cells, p = 0.0162. **h**) As in **g**), for recurrent GBMs. **i, j**) NF1-mutant vs. NF1-wildtype T-cell percentages in primary and recurrent GBMs. **k, l**) Male vs. female sex, comparing T-cell percentages in primary and recurrent GBMs, p = 0.0247. **m**) The distribution of miss-match repair (MMR) average gene expression across samples, in snRNA-seq of neoplastic cells, with the threshold used to define high- and low-expressing groups annotated. **n, o**) A significant difference in the percentage of tumor-associated T cells is seen when comparing MMR-low and MMR-high primary GBMs (p = 0.05), however, this comparison is not significant at recurrence. **p, q**) MMR gene-mutation status does not predict T-cell infiltration in primary or recurrent GBM. **r**) MMR gene-expression predicts tumor mutational burden in primary GBMs (p = 0.0302) but not in recurrent GBMs. **s, t**) IHC for CD8 in FFPE slides, representative data from 4 independent experiments with similar results. The top panels show primary GBM cases and sporadic T cells. The bottom panels show the corresponding patient-matched recurrent cases and an infiltration of CD8 + T cells into the cellular tumor. The numbers of independent samples used in boxplots are as follows, D: N = 20, E: N = 31, F: N = 4, G-L and N-R: N = 31. Boxplots in panels D-L and N-R are defined as follows, lower/upper whiskers: smallest/largest observation ≥/≤ the lower/upper hinge −/+ 1.5 times the interquartile range (IQR); lower/upper hinge: 25^th^/75^th^ percentile; center: 50^th^ percentile.

Tumor-associated lymphocytes consisted of only 0.9% and 1.7% of cells in primary and recurrent GBMs, respectively, with no significant change at recurrence in paired samples. When we performed clustering on lymphocytes (Fig. 5e and Extended Data Fig. 5a,b), the majority were identified to be in an exhausted state based on the expression of immune checkpoints (Fig. 5f). The percentage of exhausted T cells significantly increased at recurrence, at the expense of regulatory and proliferating T-cell percentages (Fig. 5g). Although T cells in total represented less than 1% of cells found in most specimens, T-cell abundance at recurrence did correlate with a significant increase in overall survival (Fig. 5h and Extended Data Fig. 5c). This result was apparently driven by 16% of recurrent cases which had levels of T-cell infiltration 2–20-fold higher than average. To identify correlates of these T-cell outliers we first assessed hypermutation status, as defined by greater than 20 mutations per megabase of DNA^21,22^. Hypermutation correlated with significantly higher levels of T-cell infiltration at recurrence when compared to either non-hypermutated recurrent cases or patient-matched primary cases (Fig. 5h and Extended Data Fig. 5d–f). While neurofibromin 1 (NF1) mutation status correlated with significantly increased innate immune-cell infiltration in primary GBMs (Extended Data Fig. 5g), this correlation did not hold in recurrent cases (Extended Data Fig. 5h). In contrast to previous reports^23^, NF1 mutations did not correlate with T-cell abundance (Extended Data Fig. 5i,j). Female sex was another correlate of increased T-cell infiltration at recurrence, but this association did not hold in primary cases (Extended Data Fig. 5k,l). When we assessed mismatch-repair (MMR) gene expression and mutation status we found that MMR expression, but not mutation status, correlated with increased T-cell infiltration in primary GBMs alone (Extended Data Fig. 5n–q). Consistent with these findings, MMR expression correlated with mutational burden only in primary GBMs (Extended Data Fig. 5r). To validate T-cell levels measured by snRNA-seq, we performed immunohistochemistry for CD8 (a marker of activated T cells) on slides from three T-cell outlier recurrent GBMs and their non-outlier patient-matched primary tumors (Fig. 5i and Extended Data Fig. 5s,t). The primary tumors had typical low levels of CD8^+^ T-cell abundance and CD8^+^ T cells were sparse, isolated and frequently confined to the perivascular space. On the other hand, the matched recurrences showed robust T-cell invasion of the cellular tumor.

### Spatial analysis yields cell-extrinsic therapeutic targets

**Fig. 6:**
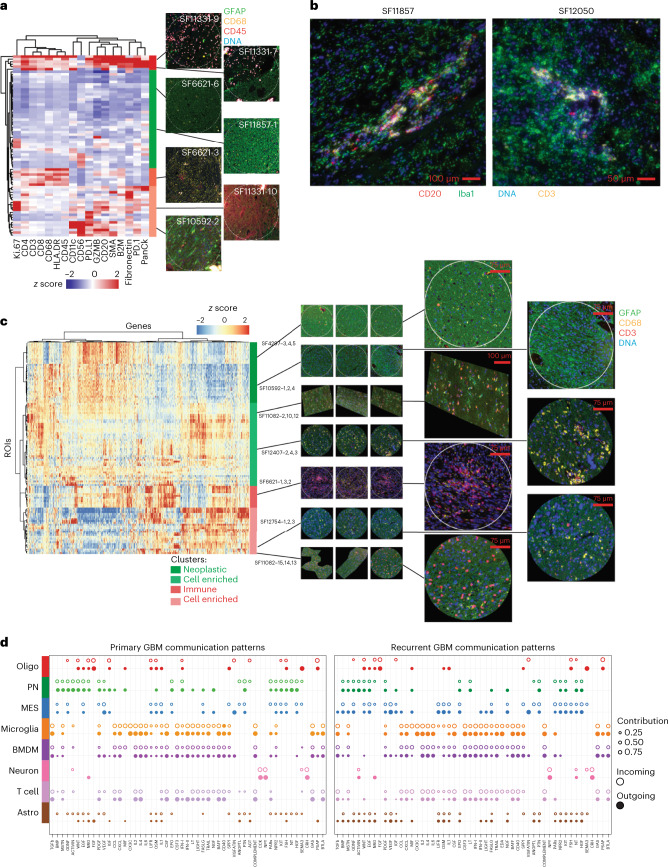
A spatial transcriptomic and proteomic atlas of human GBM through recurrence. **a**, A hierarchical clustering of SP ROIs with IF of typical ROIs (from 72 ROIs from six slides assayed) corresponding to the associated protein signatures (right). **b**, IF of two T-cell outlier cases indicating the presence of putative tertiary lymphatic structures, out of three outlier cases assayed. **c**, A hierarchical clustering of ST ROIs across genes. IF of typical ROIs (from 120 ROIs from ten slides assayed) corresponding to the associated mRNA signatures are annotated (right). **d**, Incoming and outgoing auto/paracrine signals between GBM cell types, inferred from snRNA-seq and compared between primary and recurrent GBM specimens, from *n* = 86 tumors. Receptor–agonist pairs were summarized by pathway and are annotated (bottom).

To further evaluate immune outlier cases, we profiled six formalin-fixed paraffin-embedded (FFPE) slides from three recurrent outlier cases and their matched primary cases by SP, using the Nanostring GeoMx platform (Methods). For tissue visualization, we performed immunofluorescence (IF) for glial fibrillary acidic protein (GFAP) (broadly expressed in glia), CD68 (enriched in innate immune cells), CD45 (a pan-immune marker) and DNA. Approximately 12 regions of interest (ROIs) were profiled for 17 immune-related targets per slide (Fig. 6a). Having clustered our normalized SP data, we identified regions devoid of immune infiltrates and regions expressing high levels of markers of T cells (for example CD4). We observed two types of T-cell-rich regions in the SP data, the first correlated with expression of immune checkpoints (PD-1 and PD-L1), antigen presentation/presenting-cells (CD68, CD11c, HLA-DR and B2M) and blood vessels (SMA and PanCk). The second T-cell rich cluster showed little expression of PD-1 or PD-L1. Notably, all outlier cases contained some ROIs that were enriched for B-cell markers, such as CD20, along with markers of dendritic cells (for example CD11c) and T cells (for example CD4). IF on adjacent slides for Iba, CD3 and CD20 confirmed the aggregation of B cells, T cells and monocytic-lineage cells that were consistent with the presence of tertiary lymphatic structures, for two of the three outlier cases surveyed (Fig. 6b).

**Extended Data Fig. 6:**
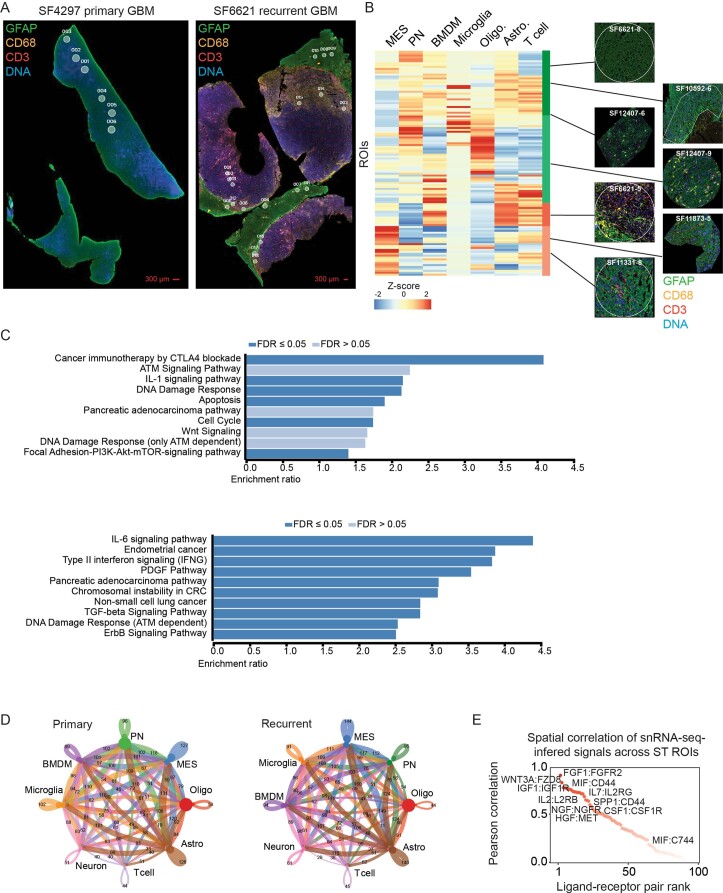
Pathway and network analysis of paracrine signaling from ST data. **a**) Representative IF images visualizing glia, innate and adaptive immune cells, performed on patient-matched primary (left) and recurrent (right) FFPE GBM specimens (from 120 ROIs from 10 slides assayed). ROIs used for in ST profiling of this specimen are annotated. **b**) A heatmap showing the relative contributions of neoplastic, glial and immune cell types inferred by deconvolving ST data using snRNA-seq signatures. IF of typical ROIs corresponding to the associated cell composition signatures are annotated on the right. **c**) (Top) a pathway enrichment analysis via WebGestalt, using Wikipathway.org pathway annotations, for genes in the first two clusters of the ST data shown in Fig. 6c. (Bottom) as above, but for the third and fourth clusters. **d**) A summary of inferred intercellular paracrine signals, based on snRNA-seq data, between different GBM cell types and compared between primary and recurrent GBM. The numbers annotated for each interaction denote the number of genes involved in the given signaling pathway. **d**) Pearson correlations across ST ROIs and samples for receptor-agonist gene pairs inferred from snRNA-seq data.

We next sought to expand our spatial analysis more broadly by performing ST on ten slides from five patient-matched primary-recurrent pairs (for example Extended Data Fig. 6a). We compared ST datasets via hierarchical clustering (Fig. 6c) and performed deconvolution analysis of the ST data based on our snRNA-seq derived signatures (Extended Data Fig. 6b and Methods). This analysis divided samples into four clusters based on two gene sets. The first two clusters were enriched for markers of proliferating tumor cells and depleted of characteristic markers of immune cells (Extended Data Fig. 6c and Supplementary Tables 10 and 11). These two clusters also had higher PN cell percentages. By contrast the third and fourth clusters overexpressed class-II interferon-signaling genes, class I and II human leukocyte antigen (HLA) and markers of innate and adaptive immune cells. We reasoned that an integrated analysis of ST and snRNA-seq data could be used to map paracrine signals in the GBM microenvironment and identify therapeutic targets. To that end, we first used CellChat to infer receptor–agonist interactions between different GBM cell types using snRNA-seq data (Methods). This approach integrates databases of agonist–receptor interactions with agonist–receptor subunit and antagonist expression levels to infer cell-type-specific signaling networks. This identified a diverse network consisting largely of secreted ligands and their receptors. We mapped differences between primary and recurrent specimens, differences between PN and MES neoplastic cell types and differences between monocytic-lineage cells based on ontogeny (Fig. 6d, Extended Data Fig. 6d and Supplementary Table 12). To validate these findings from snRNA-seq in the ST data, we computed Pearson correlations between agonist–receptor gene pairs across ROIs and samples for the hits from our CellChat analysis (Extended Data Fig. 6e and Supplementary Table 12).

**Fig. 7:**
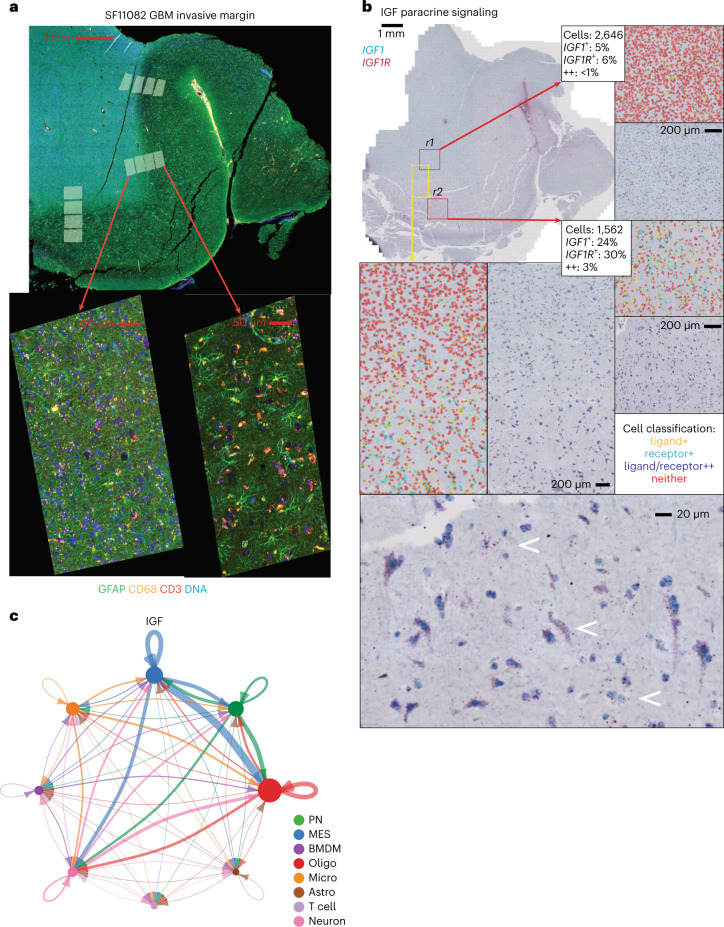
Integration of snRNA-seq and ST data identifies tumor-supportive paracrine signals with nonmalignant glia. **a**, IF in a GBM specimen used for ST, representative of *n* = 10 tumors. **b**, RNAscope on sections adjacent to **a**. Alongside are images where cells have been segmented and receptor/ligand stains quantified. Receptor mRNA is tagged red and ligand mRNA is teal. In processed images, ligand-expressing cells are yellow, receptor-positive cells are cyan, double-positive cells are purple and double-negative cells are red. RNAscope double staining for the receptor/ligand *IGF1*/*IGF1R* is shown. A window spanning the tumor-normal interface is highlighted in yellow, with a breakout showing a gradient of *IGF1* and *IGF1R* expression. Breakouts (r1 and r2) highlight sporadic *IGF1*/*IGF1R* expression in the cellular tumor and elevated *IGF1*/*IGF1R* expression in diffusely infiltrated, adjacent nonmalignant tissue. **c**, A network diagram of *IGF1*/*IGF1R* signaling from snRNA-seq, shown alongside RNAscope from the invasive edge.

**Extended Data Fig. 7:**
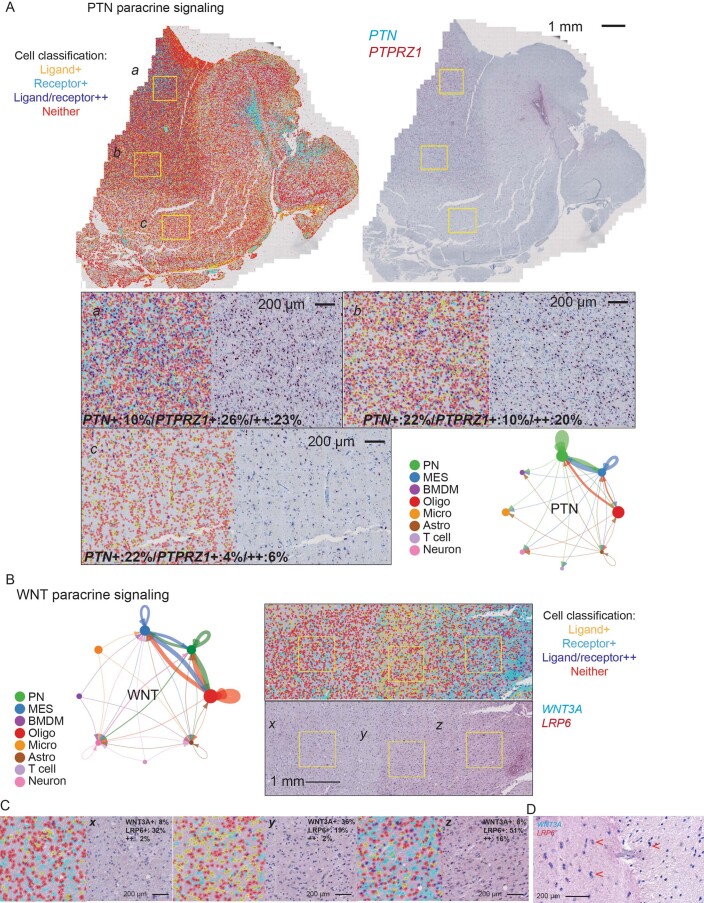
Extended RNAscope analysis. **a–c**) RNAscope on sections adjacent to Fig. 7a. Alongside are images where cells have been segmented and receptor/ligand stains quantified. Receptor mRNA is tagged red and ligand mRNA is teal. In processed images, ligand-expressing cells are yellow, receptor-positive cells are cyan, double-positive cells are purple, and double-negative cells are red. **a**) RNA-scope analysis of *PTN*/*PTPRZ1*. Double-positive cells are enriched in the cellular tumor, for example region a), *PTN*-expressing non-malignant cells are more frequent in tumor-adjacent tissue, that is **b, c**), and this gradient anticorrelates with *PTPRZ1*-expressing neoplastic cells. **b**) Gradients of WNT3A and LRP6 expression indicate the presence of both paracrine and autocrine signaling. **c**) Breakouts of sub-panels x-z) from panel B). **d**) A breakout of an interface between tumor and non-malignant tissue.

**Extended Data Fig. 8:**
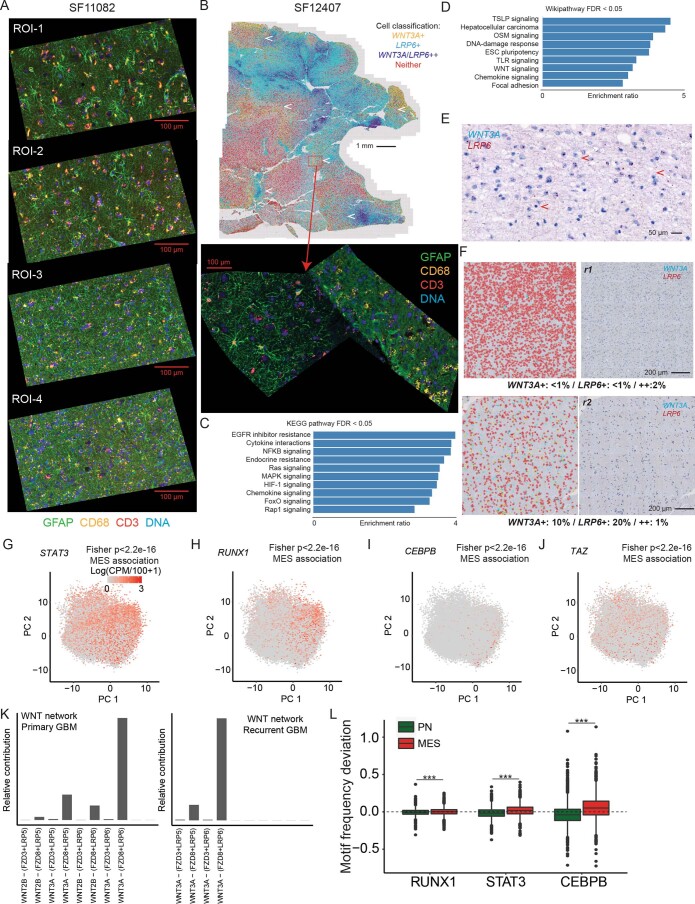
Extended data analysis. **a**) The right most ladder of ROIs shown in Fig. 7a, showing the transition from the dense cellular tumor to diffusely infiltrated adjacent non-malignant brain tissue in a primary GBM (SF11082). **b**) RNAscope profiling of *WNT3A*/*LRP6* and downstream cell classification (top) in a specimen containing interfaces between dense cellular tumor and tumor-adjacent diffusively infiltrated non-malignant tissue. The center subpanel shows adjacent tissue to the region annotated that was used for ST. Regions of putative paracrine signaling are annotated with white arrows. **c, d**) KEGG and Wikipathway gene associations that are over-represented in the ligand-receptor pairs that were inferred from snRNA-seq/ST co-analysis, and that correlate on average with progression from the cellular tumor to adjacent non-malignant tissue in SF11082. **e**) RNA-scope for *WNT3A* and *LRP6* showing interactions between *WNT3A-*/*LRP6* + , *WNT3A* + /*LRP6-*, and *WNT3A*/*LRP6* + + cells, indicating putative paracrine signaling. **f**) RNA-scope (right) and associated cell classification (left) for *WNT3A*/*LRP6* in regions *r1* and *r2* from Fig. 7b, taken from dense cellular tumor and diffusely infiltrated tumor-adjacent tissue. The frequencies of *WNT3A* + and *LRP6* + cells (but not double-positive cells) increase over 10–20 fold in regions of diffusely infiltrated non-malignant tissue (for example *r2*), compared to regions of dense cellular tumor (for example *r1*). **g–j**) Expression in snRNA-seq of N = 78,415 neoplastic cells for transcription factors implicated previously as regulators of the MES phenotype. Significance for G-J is assessed via a one-sided Fisher’s exact test. **k**) The relative contributions to the WNT signaling pathway, compared between primary and recurrent specimens. **l**) Motif frequency deviations in neoplastic cells for transcription factors implicated previously as regulators of the MES phenotype, compared between PN and MES cells. N = 14 independent samples were used. Boxplots are defined as follows, lower/upper whiskers: smallest/largest observation ≥/≤ the lower/upper hinge −/+ 1.5 times the interquartile range (IQR); lower/upper hinge: 25^th^/75^th^ percentile; center: 50^th^ percentile. A Wilcoxon rank-sum test was used. *** - adjusted p < 2.2e-16.

We next focused on signaling between neoplastic cells and nonmalignant glia, as recurrent disease often emanates from regions of diffuse infiltration and glial interactions in that niche are poorly understood. We leveraged two recurrent GBM cases (SF11082 and SF12407) where the interface between tumor and nonmalignant tissue could be discerned in FFPE slides (Fig. 7 and Extended Data Figs. 7 and 8). Many of the ligand–receptor interactions that we inferred from the above snRNA-seq/ST co-analysis were positively correlated with progression from tumor to adjacent tissue across all three serial ROI ladders in SF11082 (Fig. 7a and Extended Data Fig. 8a). Correlated genes were enriched for ontology annotations that included WNT, growth factor, cytokine and chemokine signaling (Extended Data Fig. 8c,d). We validated a tractable number of these paracrine networks in adjacent sections via RNAscope duplex in situ hybridization (RNAscope). In particular, we assessed IGF1-IGF1R, PTN-PTPRZ1, WNT3A-LRP6 and WNT2B-LRP6 coexpression in SF11082 and SF12407. We then quantified the frequencies of receptor-positive, ligand-positive and double-positive cells (Methods). For IGF and WNT pathway genes, we identified positive gradients of receptor-expressing and ligand-expressing cells as we transitioned from tumor to nonmalignant tissue (Fig. 7b,c and Extended Data Figs. 7 and 8e,f). We observed fourfold to tenfold increases in the frequencies of both receptor-expressing and ligand-expressing cells in diffusely infiltrated nonmalignant adjacent tissue when compared to regions of dense cellular tumor (Fig. 7 and Extended Data Figs. 7 and 8f). Notably, for IGF and WNT pathways, cells that were double positive for both the receptor and cognate ligand were infrequent. Instead, ligand-positive putative neoplastic cells were found adjacent to receptor-positive apparent nonmalignant glia, validating our predicted paracrine networks (Fig. 7, Extended Data Figs. 7 and 8e and Supplementary Table 12). By contrast, the PTN-PTPRZ1 RNAscope assays indicated higher levels of autocrine signaling in regions of dense tumor, evidenced by a nearly fourfold drop in double-positive cells when comparing regions in the cellular tumor to adjacent tissue. While the frequency of cells expressing *PTN* increased in the transition from tumor to adjacent tissue, the frequency of cells expressing *PTPRZ1* decreased in an anticorrelated fashion. This is consistent with the asymmetric signaling from nonmalignant to malignant glia that was predicted by our snRNA-seq/ST network analysis for PTN-PTPRZ1 (Extended Data Fig. 7a).

**Fig. 8:**
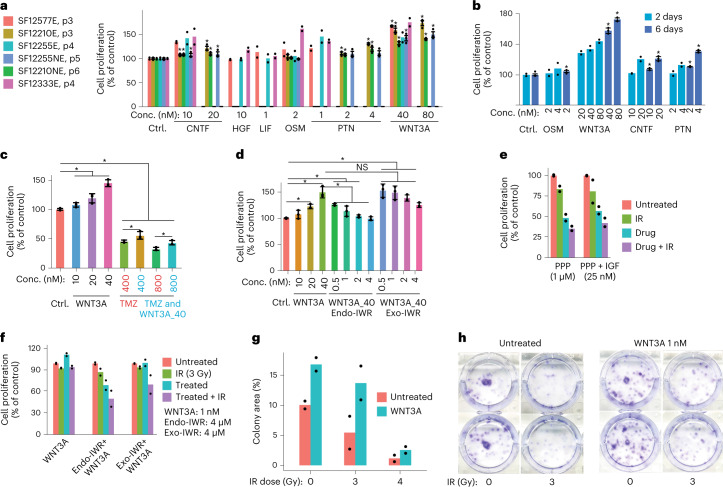
In vitro study of learned paracrine signals. **a**, Proliferation across six cell lines assayed post treatment for 6 d with recombinant proteins at varied concentrations, in *n* = 2 or *n* = 3 independent experiments per recombinant, as indicated. In **a**–**d**, where *n* = 3, a one-sided *t*-test was used (*P < 0.05) and error bars indicate ± s.d. In **a**–**g**, bar heights indicate the mean. **b**, Cell proliferation assayed after 2 and 6 d, in *n* = 2 or *n* = 3 independent experiments, respectively. **c**, WNT3A treatment significantly enhances resistance to TMZ treatment. *n* = 3 independent experiments. **d**, Co-treatment with recombinant WNT3A and a WNT3A inhibitor (Endo-IWR), but not treatment with a highly specific negative control (Exo-IWR), recovers baseline proliferation levels of treatment with WNT3A ligand. *n* = 3 independent experiments. **e**, Treatment with recombinant IGF1, with and without 3 Gy IR or an IGF inhibitor (PPP) and controls. *n* = 2 independent experiments. **f**, Treatment with WNT3A, with and without 3 Gy IR, Endo/Exo-IWR and controls. *n* = 2 independent experiments. **g**,**h**, Clonogenic assays with 1 nM WNT3A treatment, with and without 3 Gy IR and controls. *n* = 2 independent experiments. **b**–**h** utilize the cell line SF12210c1 from **a**. Source data

To assess the functional impact of these signals, we treated our panel of cell lines with recombinant IGF1, PTN and WNT3A (Fig. 8). We also tested recombinant CNTF, HGF, LIF and OSM (Methods), as these pathways had likewise been implicated in our network analysis. We found that each of these treatments significantly increased proliferation in at least some cell lines and that CNTF, PTN or WNT3A treatment robustly and consistently increased proliferation across all lines (Fig. 8a), in a dose-dependent fashion (Fig. 8b). WNT3A treatment provided a modest, but statistically significant degree of protection against TMZ chemotherapy (Fig. 8c). The baseline rate of proliferation could be recovered post WNT3A treatment via WNT3A inhibition (Fig. 8d). Moreover, WNT3A significantly enhanced colony formation and conferred resistance to IR (Fig. 8f–h).

## Discussion

We constructed a single-cell atlas of GBM under therapy, including RNA, open-chromatin and spatial readouts. Additionally, we have derived results from this resource regarding the molecular basis for MES transition, genetic correlates of the MES phenotype, the relationship between hypermutation status and T-cell infiltration, as well as cell-intrinsic and cell-extrinsic targets for therapeutic development.

We previously found that the largest source of variation in the phenotypes of proliferating cells from primary GBMs is the PN–MES axis^8^. Our meta-analysis of public and in-house snRNA-seq data (Fig. 2a–e), as well as our analysis of open-chromatin signatures (Fig. 3f–i), supports this finding and extends it to recurrent GBM. A recent study that profiled GBM stem cells found a similar gradient between what they termed developmental and injury-response states, bearing markers of the PN and MES subtypes, respectively^6^. Another recent study of GBM stem cells likewise reported a single axis of variation in phenotypes between what they call MES and non-MES cell types, which strongly correlated with our PN and MES signatures at both the level of gene expression as well as DNA methylation patterns^24^. Gangoso et al. also clearly show that a component of the MES signature is induced by immune cells and likely driven by interferon exposure.

These results dovetail with the recent findings of Schmitt et al., who used a reporter system driven by PN- and MES-specific enhancer sequences^25^. Their studies indicate that the MES phenotype is inducible, for example via exposure to TNF-α and driven by NF-κB upregulation. This is consistent with previous studies in PN GBM models^13^. The notion that the MES phenotype is inducible is supported by our analysis of the AP1 regulome (Figs. 3 and 4). This analysis shows that most MES genes are enhanced by AP1. It is well known that AP1 regulates responses to external stimuli, such as growth factors, cytokines and reactive oxygen species. We were able to induce AP1 expression and downstream MES hallmarks in vivo via treatment with IR. We also found a correlation between whole-chromosome copy-number gains on chromosomes 19 and 20 and the MES signature (Fig. 2). Taken together these results support the hypothesis that the MES phenotype is inducible yet biased by genetics. In the reporter system of Schmitt et al. the induction of a MES phenotype was reversible. Here too, we could regress MES genes via AP1 inhibition (Fig. 4). Strategies to reverse the MES phenotype would clearly be therapeutically relevant.

The basis for the MES shift in recurrent GBM is not completely understood. Multiple factors that are related to standard therapy could, in principle, contribute to this shift, for example changes in the cellular rates of division within MES and non-MES populations in response to therapy, a phenotypic shift within individual cells to a MES phenotype, preferential resistance to standard therapy by quiescent MES cells or genetic alterations that bias cells toward the MES state. There is certainly evidence in our data and from recent studies supporting all these mechanisms as factors. In our data the contribution from phenotypic shifts within individual cells was modest when measured via RNA velocity (Extended Data Fig. 3b); however, because some of these samples have been taken months after treatment pressure is removed, we cannot rule out a phenotypic shift occurring within individual cells in response to treatment playing a significant role in establishing the increased prevalence of MES cells seen at this later stage. A clear contribution to the MES shift at recurrence seems to be a marked increase in the percentage of MES cells which are cycling (Fig. 3b,c). This percentage stands in stark contrast to the strong correlation observed between the MES phenotype and quiescence in primary, treatment-naive GBM^8,9^.

Recently, Alexander et al. found that Olig2^+^ radiosensitive proliferating cells consisted of the bulk of treatment-naive PN murine gliomas^26^; however, side-population, immunohistochemistry (IHC) and scRNA-seq analysis also identified Olig2^−^/Nestin^+^ radioresistant perivascular cells that proliferated in response to radiotherapy^26^. The analysis by Alexander et al. of upstream transcription factors implicated the AP1 component FOSL2 and other genes that have been associated with the MES phenotype in previous reports^16^ and in our snRNA-seq and scATAC-seq (Figs. 3f–i and 4a–e). These findings are also consistent with the Holland laboratory’s previous report of a PN-to-MES shift in this model following radiotherapy^14^. Taken together, ours and previous studies support the premise of a quiescent stem-like cell with MES characteristics that is resistant to IR and TMZ, but that re-enters the cell cycle following therapy and becomes a driver of recurrent disease.

We observed an increase in the relative proportion of monocytic-lineage cells from the periphery at recurrence (Fig. 5a). We and others have shown that the abundances of myeloid-derived cells from peripheral blood and MES neoplastic cells correlate across patients and that these cell types co-occupy distinct tumor-anatomical niches^8,10,27^. For hypermutated cases, recurrence also correlated with a significant increase in T-cell abundance (Fig. 5h). By contrast, Hodges et al. concluded no association between tumor mutational burden (TMB) and T-cell infiltration based on IHC for PD-1, PD-L1 and CD8 in human GBMs^21^; however, while the cohort sizes interrogated for PD-1 and PD-L1 were sizable according to Hodges et al. (*n* = 94 and 189, respectively) the cohort interrogated for CD8 (*n* = 9) was less so and other pan-T-cell markers were not considered. Touat et al. tested for an association between MMR deficiency and T-cell infiltration in GBM (again via IHC) and found none, but TMB and T-cell abundance were not compared^22^. Our results most closely align with those of Wang et al.^3^, which we extend with our finding of a significant increase in T cells in high-TMB recurrences compared to recurrent cases with mid and low TMBs. TMZ treatment has been associated with hypermutation status at recurrence^28^, although hypermutation status has not been reported to convey increased survival^4^. By contrast, we found that T-cell abundance was correlated with a significant increase in survival (Fig. 5h). This seems to be driven by a cohort of outlier recurrent cases, whose T-cell abundances were two to eightfold higher than average. T cells represent just over 1% of the cellular tumor on average, so this is not high in absolute numbers; however, it indicates that (1) GBMs with a higher mutational burden are potentially more immunogenic; and (2) standard care has a treatment effect on the adaptive immune response; T-cell outlier cases are all recurrences and TMB correlates with significant increases in T-cell abundance only at recurrence (Fig. 5h and Extended Data Fig. 5d,e). T cells in most tumors were either isolated cells in a field of apparent glia or confined to a perivascular space (for example Fig. 5i). By contrast, T cells in the outlier recurrent cases robustly invade the cellular tumor (Fig. 5i and Extended Data Figs. 5s,t and 6a).

The innate immune compartment showed a remarkable lack of activation in primary GBM according to standard markers for the M1 and M2 phenotypes (Fig. 5b–d). This is consistent with previous reports^20^; however, we found significant increases in the percentages of activated innate immune cells at recurrence (Fig. 5d). Although we did not see a significant increase in the total abundance of innate immune cells in recurrent GBMs (Fig. 5a, top), we did find a significant increase in the relative abundance of innate immune cells which were classified as being derived from circulation (Fig. 5a, bottom). At the same time, the M1/M2 paradigm is overly simplistic and many patterns of non-canonical innate immune activation have been described^10^. A spectrum model may be more suitable than a bimodal M1/M2 classification^29^.

The tumor microenvironment shapes the composition of GBM neoplastic cells^30^. We modeled autocrine and paracrine signaling networks in GBM through an analysis of receptor and paired agonist expression in our snRNA-seq data (Fig. 6d, Extended Data Fig. 6d,e and Supplementary Table 12). These networks were subsequently validated via ST, RNAscope and in vitro analysis (Fig. 7 and Extended Data Fig. 7). Many of the inferred networks represent well-studied pathways in GBM: inflammation (for example interleukin-1–4 and type II interferon), immune-cell chemotaxis (for example colony-stimulating factor and CCL/CXCL) and angiogenesis (for example platelet-derived growth factor and vascular endothelial growth factor). These pathways were active in the initial disease and remained persistently active through recurrence; however, others were specifically upregulated in recurrent GBM, particularly in MES cells. For example, MES cells expressed receptors for WNT, NRG, NGF and IGF pathway genes specifically at recurrence (for example Fig. 6d, Extended Data Fig. 6d and Supplementary Table 12). This may reflect differences in microenvironment composition at recurrence. We observed a greater abundance of nonmalignant oligodendrocyte-lineage cells and innate immune cells derived from peripheral blood in recurrent specimens (Fig. 5a).

Early single-cell studies in GBM were limited to working with fresh tissue. Although these studies yielded unprecedented insights into GBM cellular composition and the tumor microenvironment, the requirement for prospective sample collection limited our statistical power as well as the types of cohorts we could assemble. Recent seminal studies demonstrated that nuclei could be efficiently extracted from archival frozen tumor specimens for single-nucleus profiling and that the resulting data were quantitative and comparable to scRNA-seq data^31^. This advance opened tissue archives to studies such as the one presented here; however, while profiling nuclear RNA gives an accurate quantification of relative gene expression there is a loss of information from a lack of mitochondrial RNA in snRNA-seq. In addition to the information about metabolism that can be gleaned from mitochondrial RNA^24^, expressed mitochondrial mutations can be used for single-cell phylogenetics^8,32^. More generally, our droplet-based approach yields 3′-enriched coverage that is intended only for quantification of gene expression. Single-cell alternative splicing, an increasingly recognized contributor to tumor immunogenicity^33^, and single-cell analysis of expressed mutations are not optimal in these data. Last, epigenetic regulation is increasingly understood as an important lens through which therapeutically relevant processes in GBM can be understood, for example the MES shift at recurrence (Figs. 3 and 4), stemness and tumorigenicity^34^, oncogene amplification^35^ and sex differences^36^, to name a few. Single-cell epigenetic analyses of longitudinal brain tumor specimens are lacking. Given the plethora of new modalities available^37,38^, the rationale for doing these studies is strong. The resource generated by this study broadly informs disparate aspects of glioma biology. Further work will be required to functionally test many of the hypotheses generated from this atlas.

## Methods

### Ethical approval

Study protocols and sample use were approved by the University of California, San Francisco (UCSF) Institutional Review Board. All clinical samples were analyzed in a de-identified fashion. All experiments were carried out in conformity to the principles set out in the Declaration of Helsinki as well as the Department of Health and Human Services Belmont Report. Informed written consent was provided by all patients.

### Tumor tissue acquisition

We obtained fresh-frozen and FFPE tissue specimens from patients undergoing surgical resection for glioma at UCSF. De-identified samples were provided by the UCSF Neurosurgery Tissue Bank.

### Statistics and reproducibility

No statistical method was used to predetermine sample size. All available GBM specimens in the UCSF Brain Tumor Center Tissue Bank were profiled, representing decades of biobanking at UCSF. No data were excluded from the analyses. Randomization and blinding was used for all in vitro and in vivo experiments. The Wilcoxon signed-rank test for paired samples was used to compare percentages of cell types between primary and paired recurrent specimens. As the Wilcoxon test is nonparametric, we did not formally test for normality of the data. A log-rank test was used to assess significance in Kaplan–Meier survival analysis. A two-sided likelihood-ratio test between hurdle models was used to assess differential gene expression between single-cell datasets and were adjusted for multiple hypothesis testing via the Benjamini–Hochberg method. Fisher’s exact test was used to test for genotype–phenotype associations.

### Nuclei isolation

For snRNA-seq, nuclei were extracted from frozen tissues following the ‘Frakenstein’ protocol developed by L. Martelotto, Melbourne, Centre for Cancer Research, Victorian Comprehensive Cancer Centre and available from 10x Genomics (https://community.10xgenomics.com/t5/Customer-Developed-Protocols/ct-p/customer-protocols). For snATAC-seq, frozen tissues were digested mechanically in a Dounce grinder with 500 µl of lysis buffer (Sigma). The lysate was strained through a 40-μm strainer, pelleted, washed and resuspended in 500 µl nuclei wash buffer (10x Genomics). Nuclei were subsequently purified via centrifugation in a sucrose-based density gradient, pelleted, washed and resuspended in tagmentation buffer (10x Genomics).

### Cell derivation, culture and in vitro viability assays

Fresh tumor tissues were dissociated mechanically with a scalpel and then enzymatically (32 mg collagenase IV, 10 mg deoxyribonuclease I, 20 mg soybean trypsin inhibitor and 10 ml DPBS) at 37 °C under rotation for 15 min. Tissue lysate was further dissociated via pipetting and incubated at 37 °C for another 15 min with rotation. The lysate was filtered through a 70-μm strainer then a 40-μm strainer, washed twice and resuspended in RBC lysis buffer for 5 min. Cells were spun down, washed and resuspended in culture medium (DMEM/F12 GlutaMAX, 0.5% N2, 0.5% B27 without vitamin A, 1% antibiotic-antimycotic, 0.5% NEAA, 20 ng ml^−1^ epidermal growth factor and 20 ng ml^−1^ fibroblast growth factor). Cells were cultured in Matrigel-coated plates at 37 °C and 5% CO_2_.

Where indicated, cell viability was assessed via AlamarBlue (Invitrogen) reduction. Briefly, cells were seeded in 96-well plates at a density of 5,000 cells per well with DMEM/F12 complete medium and held overnight at 37 °C and 5% CO_2_. The medium was then aspirated and test compounds diluted in culture medium were administered at the reported concentrations. After culture for the reported time periods, the cells were washed with PBS and resuspended in 9 ml of culture medium plus 1 ml of AlamarBlue (Invitrogen). The plate which was then incubated for 4 h. Cell viability was assessed via absorbance using a microplate reader and compared to vehicle-treated control wells. In other experiments, proliferation was measured by trypan blue cell counting via a Countess II.

For WNT3A inhibition endo-IWR 1 (R&D Sys) was used. This is a small molecule inhibitor of Axin turnover resulting in stabilization of the β-catenin and suppression of Wnt signal transduction. The diastereomeric form of IWR 1, exo-IWR 1, which exhibits decreased Axin stabilizing activity compared to endo-IWR 1 was used as a control.

### Clonogenic assays

Cell we seeded at a density of 600 cells per well in the middle wells of six-well plates that were coated with 2% Cultrex TM BME (R&D Sys A1569601). Cells were incubated at 37 °C and 5% CO_2_ for 2 h to allow attachment. Subsequently, plates were exposed to 3 or 4 Gy of gamma radiation (JL Shepherd & Associates) in rotation function without plate cover via a cesium-137 source emitting at a fixed dose rate of 2.46 Gy min^−1^. After 10 d, colonies were fixed using 6% glutaraldehyde diluted in DPBS and stained with 1 ml of Crystal Violet. ImageJ (v.1.51 h) was used for automated colony counting.

### Multiplex immunofluorescence

FFPE tumor sections were profiled by multiplex immunofluorescence using a Discovery XT autostainer (Ventana Medical Systems) with appropriate controls. Antibodies used were CD3 (Leica, Clone LN10, 1:100 dilution), CD20 (Leica, Clone L26, 1:200 dilution) and Iba1 (Wako Chemicals, 019-19741, 1:500 dilution).

### Dual RNAscope

FFPE sections were evaluated by dual RNAscope chromogenic in situ hybridization assay for the expression of ligand–receptor pairs using Advanced Cell Diagnostics probes specific for PTN (838191) and PTPRZ1 (584789-C2), WNT2B (453369) or WNT3A (429439) and LRP6 (custom probe, C2) and IGF1 (313039) and IGF1R (415819-C2). The RNA Probe PPIB (313909) and dapB (312039) were used as positive and negative control probes, respectively. Cell segmentation, classification and staining quantification were performed via QuPath (v.0.3.2).

### Murine IR assay

All animal experiments were conducted in compliance with protocols approved by the Institutional Animal Care and Use Committee at UCSF, following the National Institutes of Health (NIH) guidelines for animal care. The UCSF Institutional Animal Care and Use Committee maximal tumor burden of 20 mm in any direction was not exceeded. Ten-week-old C57BL/6J female mice were purchased from the Jackson Laboratory (000664) and housed in the UCSF animal facility 1 week before tumor transplantation at temperatures of 65–75 °F (~18–23 °C) with 40–60% humidity and a 14–10-h light–dark cycle. An aliquot of 1,600 SB28 cells^19^ were injected into the right frontal cortex at the coordinate Bregma, AP +2.0 mm, ML +2.0 mm and DV −2.0 mm in a cohort of six mice. All mice developed tumors, based on bioluminescence imaging. On days 10, 12 and 14 after tumor transplantation, 5 Gy head-only irradiation was given to three randomly chosen tumor-bearing animals as previously described^39^. Endpoints were determined by weight loss and neurological symptoms. Upon euthanasia, brain tumors were quickly dissected after perfusion (cold 1× PBS) and snap frozen in liquid nitrogen, then stored at −80 °C before use.

### Spatial transcriptomics and proteomics assays

For the ST assay, FFPE tissue blocks were reviewed for tumor purity and ten 5-µm sections were cut by the UCSF Neurosurgery Tissue Core. Slides were baked at 37 °C overnight and then deparaffinized, rehydrated, antigen-retrieved for 20 min at 100 °C and digested with proteinase-K 0.1 µg ml^−1^ for 15 min in a Leica BOND-RX. Samples were post-fixed in neutral-buffered formalin for 10 min and hybridized to the Cancer-Transcriptome Atlas (>1,800 targets) UV-photocleavable barcode-conjugated RNA in situ hybridization probe set overnight. Samples were washed to remove off-target probes and counterstained with morphology markers for 2 h. The morphology markers consisted of 1:25 dilution SYTO13 (Invitrogen), 1:100 dilution anti-CD3 Alexa Fluor 647 (UMAB54, Origene), 1:200 dilution anti-CD68-Alexa Fluor 594 (KP1, Santa Cruz) and 1:400 dilution anti-GFAP-Alexa Fluor 488 (GA5, Invitrogen). IF imaging, ROI selection, spatially indexed barcode cleavage and collection were performed on a GeoMx Digital Spatial Profiling instrument (NanoString). Approximately 12 ROIs were collected per sample. Photoreleased GeoMx DSP oligonucleotide tags containing RNA IDs and a unique molecular identifier were collected from each ROI. After PCR with dual-indexing Illumina i5 and i7 primers, the library was purified with AMPure XP beads (Beckman Coulter), quantitated with a Qubit (Themo Fisher Scientific) and quality was checked with a Bioanalyzer (Agilent). Paired-end sequencing was performed on NextSeq 550 and NextSeq 2000 instruments.

Similarly, for the SP assay, FFPE tissue blocks were reviewed for tumor purity and six 5-µm sections were cut by the UCSF Neurosurgery Tissue Core. Slides were baked at 37 °C overnight, deparaffinized, rehydrated, antigen-retrieved in a pressure cooker for 15 min at 100 °C at high pressure. Samples were then incubated overnight with the GeoMx Immune Cell Profiling Protein Core antibodies (NanoString) containing UV-photocleavable barcode-conjugated antibodies against 17 targets and 6 control targets. At the same time, the samples were incubated with morphology antibodies consisting of SYTO83 (100 nM final concentration), 1:200 dilution anti-CD68-Alexa Fluor 594 (clone KP1), 1:200 dilution anti-CD45-Alexa Fluor 647 (clone 2B11 + PD7/26) and 1:400 dilution anti-GFAP-Alexa Fluor 488 (clone GA5). IF imaging, ROI selection, spatially indexed barcode cleavage and collection were performed on a GeoMx Digital Spatial Profiling instrument (NanoString) by GENEWIZ. Approximately ten ROIs were collected per sample. The resulting photocleavable barcode tags were subsequently detected and counted using an nCounter Prep Station and Digital Analyzer (NanoString).

### 10x Genomics-based snRNA-seq/snATAC-Seq

Single-nucleus capture, reverse transcription, cell lysis and library preparation for snRNA-seq were performed on the 10x Genomics platform as per manufacturer’s protocol. Approximately 15,000 nuclei were loaded per capture. For snATAC-seq assay, tagmentation, nuclei capture and library prep were likewise performed via the 10x Genomics platform as per manufacturer’s protocol. Sequencing was performed on an Illumina NovaSeq with 10x Genomics recommended parameters.

### SnRNA-seq data preprocessing

The preprocessing of snRNA-seq data was performed as described previously^8^. We utilized CellRanger (v.3.0.2) for alignment and gene expression quantification, following the guidelines from the CellRanger website (https://support.10xgenomics.com/single-cell-gene-expression/software/pipelines/latest/advanced/references#premrna). We filtered cells that have >2.5% mitochondrial read counts and <200 expressed genes. DoubletFinder (v.2.0.2)^40^ was used to remove doublets and was run using the first ten PCs and default parameters.

### Copy-number variation analysis of snRNA-seq and snATAC-seq

CONICSmat (v.1.0) was used to assess the presence/absence of somatic CNVs in 10x snRNA-seq data^41^. We retained CNVs with a CONICSmat likelihood-ratio test <0.05 and a difference in Bayesian Criterion >50. For each CNV we used a cutoff of posterior probability >0.5 in the CONICSmat mixture model to infer the presence/absence of that CNV in a given cell. The presence/absence of somatic CNVs in 10x snATAC-seq data was likewise estimated with CONICSmat. Here, the gene activity of cells generated by snapATAC (v.1.0.0)^42^ was used as input to perform CNV analysis.

### Exome sequencing and copy-number variant identification

The Targeted DNA Seq Library Reagent kit was used for exome capture on tumor samples for selected patients. Libraries were sequenced on an Illumina-HiSeq 4000 using 150-bp paired-end reads. Reads were first trimmed and filtered with TrimGalore v.0.6.5 (parameter, −*q* = 30) (https://www.bioinformatics.babraham.ac.uk/projects/trim_galore/) and Cutadapt v.3.4 (ref. ^43^). The quality control passed reads were mapped to the human Grch38 genome with BWA and only uniquely matched paired reads were used for analysis^44^. PicardTools (http://broadinstitute.github.io/picard/) and the GATK toolkit carried out quality score recalibration, duplicate removal and realignment around indels^45^. CNVs were inferred with CNVkit v.0.9.6 (segment function, *P* value threshold <1 × 10^−3^)^46^.

### Dimensionality reduction, clustering and cell-type classification for snRNA-seq

The snRNA-seq data were processed with Seurat v.3 (ref. ^47^). Data were normalized via the LogNormalize method with scale.factor of 10,000 using the NormalizeData function. Highly variable genes were identified via Seurat using the mean.var.plot method with default parameters. Based on these genes, a PCA was performed and the first 15 PCs were retained for clustering and visualization via *t*-SNE. Neoplastic cells were separated from non-neoplastic cells based on the presence of CNVs. For neoplastic cells, MES and PN cell-type labels were assigned via ELSA, an ensemble-learning approach that has been trained on historical data^8,11^. For non-neoplastic cells, cell clusters were identified via the ‘FindClusters’ function via the Louvain algorithm with the resolution parameter of 0.52. Cluster-specific genes were identified via the FindAllMarkers function in Seurat v.3 with Rstudio running R v.3.6.0 (ref. ^47^) via a MAST test and used to assign cell-type labels.

### Multiple-factor analysis

To perform MFA, we obtained 10x scRNA-seq data from Neftel et al.^9^ (GSE131928) and Couturier et al.^7^ (EGAS00001004422). Cells with more than 5% mitochondrial read counts were filtered for both datasets and cells with at least 200 expressed genes were retained for analysis. The gene matrix for each dataset was filtered to only retain the highly variable genes, identified via the FindVariableFeatures function of Seurat v.3 with selection.method ‘mvp’. This was applied to each dataset separately. Then genes that were identified as variable in at least two datasets were used for the MFA (1,411 genes in total). The FactoMiner package^48^ was used to perform MFA. The contributions of each dataset to total variance explained, as well as the genes’ qualities of representation by dimensions one and two (cos^2^), was computed via the fviz_contrib function from the factoextra package^49^.

### Single-nucleotide variant calling in snRNA-seq and UCSF500 genotypes

The bam file of 10x snRNA-seq data generated via CellRanger was used to perform single-nucleotide variant calling by pooling reads by patient and running the GATK RNA-seq best-practices pipeline (https://software.broadinstitute.org/gatk/best-practices/workflow?id=11164). Variant assignments in single cells were then assessed via the VarTrix (v.1.0) tool (https://github.com/10xgenomics/vartrix). Variants were annotated with the Annovar software package^50^. The UCSF500 mutation panel is a clinical assay that uses an amplicon sequencing-based genotyping approach that compares a tumor tissue sample and a patient-matched blood control. For the mutation calling of UCSF500, reads were mapped to the human genome reference with BWA^44^. PicardTools (http://broadinstitute.github.io/picard) and the GATK toolkit^45^ carried out quality score recalibration, duplicate removal and realignment around indels. Somatic single-nucleotide variants were detected with MuTect (https://www.broadinstitute.org/cancer/cga/mutect) for each tumor–control pair. The mutations were annotated with the Annovar software package.

### RNA velocity analysis

RNA velocities were computed via scVelo using default parameters^51^. MES neoplastic cells from recurrent GBMs were used as input. SnRNA-seq data were filtered and normalized via scvelo.pp.filter_and_normalize with parameter min_shared_counts = 20. The first 30 PCs were used to compute moments for velocity estimation via scvelo.pp.moments. The terminal states and latent time of cycling cells were computed via scvelo.tl.latent_time function included in scVelo. The putative driver genes of transcriptional changes were systematically identified by high likelihoods in the dynamic model. In particular, the top 300 highest-likelihood genes were used to generate a heat map via the scvelo.pl.heatmap function. Genes that showed a Pearson correlation with pseudotime of .1 or higher at an adjusted *P* value^52^ of *q* < 0.05 were used for Gene Ontology analysis via WebGestalt using the WikiPathway reference (Fig. 3e).

### Monocytic cell classification and analysis

To classify the activation status of monocytic-lineage cells, signature genes of classically (M1: *CCL2/3/4/5/8*, *CCR7*, *CD74*, *CSF2*, *CXCL10*, *HLA-DRA*/*B*, *IFNG*, *IL1B*, *IL1R1*, *IL6*, *INOS*, *IRF5*, *NFKB1*, *TLR2*/*4* and *TNF*) and alternatively activated (M2: *ARG1*, *CD74*, *CCL1/17/22/*, *CXCL16*, *CXCR4*, *HLA-DRA*/*B*, *IL10*, *IL4*, *IRF4*, *MRC1*, *NFKB1*, *TGFB1* and *TNF*) phenotypes were aggregated from previous reports^53–57^. The M1 and M2 score for each cell was calculated via AddModuleScore function with the above M1/M2 signatures as input. This routine compares average signature levels to a data-driven background distribution. Cells were assigned M0 status if the M1 and M2 signature scores were both less than 0.0. Otherwise, activation status was determined by the higher of the M1 and M2 signature scores.

### T-cell phenotype classification and analysis

We used Seurat to perform dimensionality reduction, clustering and visualization for T cells. In particular, data were normalized via the NormalizeData function using the LogNormalize method. Highly variable genes were identified via the FindVariableFeatures function using the mvp method (mean.cutoff of c(0.1, 8) and dispersion.cutoff = c(1, Inf)) and other parameters set to default values. PCA was performed based on these genes. The first 15 PCs were retained for clustering via a *k*-nearest neighbor graph and visualized via *t*-SNE. A heat map was generated via DoHeatmap with the average expression calculated with the AverageExpression function. A boxplot of different T-cell types was generated via ggplot with geom_boxplot in R v.3.6.0.

### snATAC-seq data processing and analysis

The CellRanger ATAC software (v.1.1.0) was used for read alignment, deduplication and identifying transposase cut sites (https://support.10xgenomics.com/single-cell-atac/software/pipelines/latest/algorithms/overview). The output matrix of CellRanger was further processed via the snapATAC package (https://github.com/r3fang/SnapATAC)^42^. We selected the highest quality barcodes for each case based on two criteria: (1) number of filtered fragments >1,000; and (2) fragments in promoter ratio >0.2 for the case. Clustering was performed using Seurat v.3 SNN-graph clustering via the ‘FindClusters’ routine, with gene body-accessibility scores generated by the snapATAC package as input. Transcription factor motif frequency deviations from a data-driven background model were calculated via the computeDeviations function in chromVAR (v.1.6.0) with default parameters^58^, using only neoplastic cells as input. Differential motif deviances were computed via a *t*-test and controlled for multiple hypothesis testing via fdrtool^52^. Differentially accessible regions, peaks and motif enrichments on differential peaks (relative to a genome-wide background) were computed using snapATAC’s ‘findDAR’, ‘runMACSForAll’ and ‘runHomer’ respectively, run with default parameters. Heat maps of differential peaks were created in deepTools v.3.4.0 (ref. ^59^).

### Cell–cell communication analysis

The CellChat package was used to assess cell–cell communication via interaction-network analysis^60^. A Seurat object was used as input for CellChat following their standard protocol as described in https://github.com/sqjin/CellChat. Circle plots and dot plots were generated via netVisual_aggregate, vertex.size = groupSize and netAnalysis_dot resp. Data from primary GBM cases were processed separately from data from recurrent cases and compared a posteriori.

### Data processing for spatial transcriptomics and proteomics

The SP data were normalized by ROI surface area. The ST data were normalized against the 75th percentile of signal (Q3 normalization). Multi-subject single-cell deconvolution was used for deconvolution of the ST data based on cell-type signatures determined from snRNA-seq data used as input^61^. Heat maps were generated via pheatmap package (https://cran.r-project.org/web/packages/pheatmap/index.html). To validate putative ligand–receptor interactions via ST, the expression of each ligand–receptor pair obtained from CellChat were used to calculate a Pearson’s correlation coefficient across ROIs. These were adjusted for multiple hypothesis testing via fdrtool^52^. Representative IF images for specific pathways were obtained based on sorting for correlation and then for expression of ligand–receptor pairs in that pathway.

### Survival analysis

Survival analysis was conducted in R. Samples were divided into two groups T-cell high and T-cell low based on T-cell percentage at a cutoff of 1%. Kaplan–Meier plots of overall survival and elapsed days to recurrence for recurrent GBM were generated via ggsurvplot function in Survminer package. *P* values were calculated via a log-rank test.

### Reporting summary

Further information on research design is available in the Nature Portfolio Reporting Summary linked to this article.

## Supplementary information

Reporting Summary

Supplementary Tables Supplementary Tables 1–12

## Data Availability

The study data, in the form of raw sequenced reads, are available from the European Genome–phenome Archive repository (https://www.ebi.ac.uk/ega/home), accession EGAS00001004909. Spatial image data, processed expression and peak Supplementary Tables are available from the Gene Expression Omnibus repository (https://www.ncbi.nlm.nih.gov/geo/), accession code GSE174554. Previously published scRNA-seq data that were re-analyzed here are available from https://github.com/mbourgey/scRNA_GBM and from the Gene Expression Omnibus, accession code GSE131928. All other data supporting the findings of this study are available from the corresponding author on reasonable request. Source data are provided with this paper.

## Source data

Source Data Fig. 4 Statistical source data.
Source Data Fig. 8 Statistical source data.
Source Data Extended Data Fig. 4 Statistical source data.

## Code availability

Custom code used is available from GitHub at https://github.com/linwang6/Longitudinal_glioblastoma_paper.
