# Supplementary material for: A single-cell atlas of glioblastoma evolution under therapy reveals cell-intrinsic and cell-extrinsic therapeutic targets
Source: Nat Cancer. 2022 Dec 20;3(12):1534–52. doi: 10.1038/s43018-022-00475-x (PMC9767870; doi:10.1038/s43018-022-00475-x)
Supplement: Supplementary file 1 — Reporting Summary [file 43018_2022_475_MOESM1_ESM.pdf]

nature portfolio

Corresponding author(s): Aaron Diaz

Last updated by author(s): 7/5/2022

Reporting Summary

Nature Portfolio wishes to improve the reproducibility of the work that we publish. This form provides structure for consistency and transparency in reporting. For further information on Nature Portfolio policies, see our Editorial Policies and the Editorial Policy Checklist.

Statistics

For all statistical analyses, confirm that the following items are present in the figure legend, table legend, main text, or Methods section.

n/a	Confirmed	
☐	☒	The exact sample size (*n*) for each experimental group/condition, given as a discrete number and unit of measurement
☐	☒	A statement on whether measurements were taken from distinct samples or whether the same sample was measured repeatedly
☐	☒	The statistical test(s) used AND whether they are one- or two-sided<br>*Only common tests should be described solely by name; describe more complex techniques in the Methods section.*
☐	☒	A description of all covariates tested
☐	☒	A description of any assumptions or corrections, such as tests of normality and adjustment for multiple comparisons
☐	☒	A full description of the statistical parameters including central tendency (e.g. means) or other basic estimates (e.g. regression coefficient) AND variation (e.g. standard deviation) or associated estimates of uncertainty (e.g. confidence intervals)
☐	☒	For null hypothesis testing, the test statistic (e.g. *F*, *t*, *r*) with confidence intervals, effect sizes, degrees of freedom and *P* value noted<br>*Give P values as exact values whenever suitable.*
☒	☐	For Bayesian analysis, information on the choice of priors and Markov chain Monte Carlo settings
☒	☐	For hierarchical and complex designs, identification of the appropriate level for tests and full reporting of outcomes
☐	☒	Estimates of effect sizes (e.g. Cohen's *d*, Pearson's *r*), indicating how they were calculated

Our web collection on statistics for biologists contains articles on many of the points above.

Software and code

Policy information about availability of computer code

Data collection	No software was used for data collection.
Data analysis	The following software were used for data analysis:<br>CellRanger v3.0.2 10x Genomics http://10xgenomics.com/<br>CellRanger ATAC v1.1.0 10x Genomics http://10xgenomics.com/<br>Seurat v3 (Stuart et al., 2019) https://github.com/satijalab/seurat<br>DoubletFinder v2.0.2 (McGinnis et al., 2019) https://github.com/chris-mcginnis-ucsf/DoubletFinder<br>ELSA v1 (Wang et al., 2020) https://github.com/diazlab/ELSA<br>CONICSmat v1 (Müller et al., 2018) https://github.com/diazlab/CONICS/tree/master/CONICSmat<br>SnapATAC v1.0.0 (Fang et al., 2019) https://github.com/r3fang/SnapATAC<br>FactoMineR v2.6 (Lê et al., 2008) https://github.com/cran/FactoMineR<br>Factoextra v1.0.7 (Kassambara and Mundt, 2020) https://github.com/kassambara/factoextra<br>GATK v4.2 (McKenna et al., 2010) https://github.com/broadinstitute/gatk<br>Vartrix v1.0 GitHub https://github.com/10xgenomics/vartrix<br>BWA v0.7.17 (Li and Durbin, 2009) https://github.com/lh3/bwa<br>PicardTools v2.27.5 GitHub http://broadinstitute.github.io/picard/<br>MuTect v2 GitHub https://github.com/broadinstitute/mutect<br>Annovar v2019Dec03 (Wang et al., 2010) https://annovar.openbioinformatics.org/en/latest/<br>Scvelo v0.2.5 (Bergen et al., 2020) https://github.com/theislab/scvelo<br>ChromVAR v1.6.0 (Schep et al., 2017) https://github.com/GreenleafLab/chromVAR<br>CellChat v1 (Jin et al., 2021) https://github.com/sqjin/CellChat

MuSiC v1 (Wang et al., 2019b) https://github.com/xuranw/MuSiC
R v3.6.0 (R Core Team (2020), 2020) https://www.r-project.org/
Rstudio v1.3.1073 (RStudio Team, 2015) https://www.rstudio.com/
ggplot2 v3.3.6 (Wickham, 2016) https://cran.r-project.org/web/packages/ggplot2/index.html
Python v3.7 (Python Core Team, 2015) https://www.python.org/
Survminer v0.4.9 (Kassambara et al., 2018) https://cran.r-project.org/web/packages/survminer/index.html
TrimGalore v0.6.5 GitHub https://github.com/FelixKrueger/TrimGalore
CNVkit v0.9.6 (Talevich et al., 2016) https://github.com/etal/cnvkit
Cutadapt v3.4 (Martin et al., 2011) https://github.com/marcelm/cutadapt
QuPath v0.3.2 (Bankhead, 2017) https://qupath.github.io/
Fdrtool v1.2.17 (Strimmer, 2008) https://cran.r-project.org/web/packages/fdrtool/index.html
MAST v1.21.3 (Finak, 2015) https://rglab.github.io/MAST/
WebGestalt v2019 (Liao, 2019) http://www.webgestalt.org/

For manuscripts utilizing custom algorithms or software that are central to the research but not yet described in published literature, software must be made available to editors and reviewers. We strongly encourage code deposition in a community repository (e.g. GitHub). See the Nature Portfolio guidelines for submitting code & software for further information.

Data

Policy information about availability of data

All manuscripts must include a data availability statement. This statement should provide the following information, where applicable:

- Accession codes, unique identifiers, or web links for publicly available datasets
- A description of any restrictions on data availability
- For clinical datasets or third party data, please ensure that the statement adheres to our policy

The study data, in the form of raw sequenced reads, are available from the European Genome-phenome Archive repository (https://www.ebi.ac.uk/ega/home), accession EGAS00001004909. Spatial image data, processed expression and peak tables are available from the Gene Expression Omnibus repository (GEO, https://www.ncbi.nlm.nih.gov/geo/), accession GSE174554. Previously published scRNA-seq data that were re-analyzed here are available from https://github.com/mbourgey/scRNA_GBM and from GEO, accession GSE131928. Source data have been provided as Source Data files. All other data supporting the findings of this study are available from the corresponding author on reasonable request.

Human research participants

Policy information about studies involving human research participants and Sex and Gender in Research.

Reporting on sex and gender	Biological sex was determined based on self reporting and included as a co-variate for analysis where relevant. N=57 specimens from female subjects and N=53 specimens from male subjects were used in this study. For N=1 there was no sex information available.
Population characteristics	Specimens used in this study were derived from primary-untreated and recurrent tumor specimens. At the time of the first recurrence all patients had been treated only with standard-of-care therapy: temozolomide, ionizing radiation, and surgical resection. The ages of subjects from which specimens were derived ranged from 24-83. All cases were diagnosed as grade 4 glioblastomas.
Recruitment	This is a retrospective study of archival specimens. There was no prospective recruitment. All viable tumor specimens collected by the UCSF Brain Tumor Center Biorepository, for which informed written consent had been obtained, were used in this study.
Ethics oversight	Study protocols were approved by the UCSF Institutional Review Board. All clinical samples were analyzed in a de-identified fashion. All experiments were carried out in conformity to the principles set out in the WMA Declaration of Helsinki as well as the Department of Health and Human Services Belmont Report. Informed written consent was provided by all patients.

Note that full information on the approval of the study protocol must also be provided in the manuscript.

Field-specific reporting

Please select the one below that is the best fit for your research. If you are not sure, read the appropriate sections before making your selection.

☒ Life sciences ☐ Behavioural & social sciences ☐ Ecological, evolutionary & environmental sciences

For a reference copy of the document with all sections, see nature.com/documents/nr-reporting-summary-flat.pdf

Life sciences study design

All studies must disclose on these points even when the disclosure is negative.

Sample size	N=111 specimens were used in this study. This number was chosen to obtain the largest cohort possible, as all viable tumor specimens

Sample size	collected by the UCSF Brain Tumor Center Biorepository (for which informed written consent had been obtained) were used in this study. This represents decades of biobanking at UCSF and is consistent with sample sizes in contemporary studies.
Data exclusions	No data were excluded.
Replication	We replicated core findings obtained from snRNA-seq (N=86 independent experiments) via bulk RNA-seq (N=35 independent experiments). All in vitro or in vivo experiments were performed in duplicate or triplicate as indicated. All attempts at replication were successful.
Randomization	Cultured cells or mice were randomly assigned to treatment or control groups.
Blinding	Investigators were blinded to group allocation in all in vitro and in vivo experiments.

Reporting for specific materials, systems and methods

We require information from authors about some types of materials, experimental systems and methods used in many studies. Here, indicate whether each material, system or method listed is relevant to your study. If you are not sure if a list item applies to your research, read the appropriate section before selecting a response.

Materials & experimental systems

n/a	Involved in the study	
☐	☒	Antibodies
☐	☒	Eukaryotic cell lines
☒	☐	Palaeontology and archaeology
☐	☒	Animals and other organisms
☒	☐	Clinical data
☒	☐	Dual use research of concern

Methods

n/a	Involved in the study	
☒	☐	ChIP-seq
☒	☐	Flow cytometry
☒	☐	MRI-based neuroimaging

Antibodies

Antibodies used	CD3 Leica, Clone LN10 cat#CD3-565-L-CE<br>CD20 Leica, Clone L26 cat#CD20-L26-L-CE<br>Iba1 Wako Chemicals cat#019-19741<br>CD3 Alexa Fluor 647 Origene, Clone UMAB54 cat#UM500048<br>CD68-Alexa Fluor 594 Santa Cruz, Clone KP1 cat#sc-20060<br>CD68-Alexa Fluor 594 Invitrogen, Clone KP1 cat#MA5-13324(Thermo)<br>CD45-Alexa Fluor 647 Invitrogen, Clone 2B11+PD7/26 cat#MA5-18142(Thermo)<br>GFAP-Alexa Fluor 488 Invitrogen, Clone GA5 cat#53-9892-82(Thermo)
Validation	All antibodies were validated by the manufacturers. As stated on their respective websites, antibodies undergo two-part testing, including target specificity verification (via at least one of: knockout, knockdown, independent antibody validation, cell treatment, neutralization, peptide array, SNAP-ChIP, IP-MS) and functional application validation (via at lease one of: Western blot, flow cytometry, ChIP, IF, IHC).

Eukaryotic cell lines

Policy information about cell lines and Sex and Gender in Research

Cell line source(s)	Five human glioma cell lines were derived in house: SF12255E, SF12255NE, SF12577E, SF12210E, SF12333E. The SB28 murine glioma cell line was a gift from the Okada lab (UCSF). The Okada lab derived the SB28 cell line from a spontaneous glioma induced in a neonatal C57BL/6 mouse via ectopic expression of DNA plasmids which were mixed with in vivo compatible DNA transfection reagent, In vivo-JetPEI (Polyplus Transfection, New York, NY): pT2/C-Luc//PGK-SB100 (0.06 µg/mouse), Sleeping beauty transposon (SB)-flanked pT2/CAG-NRasV12 (0.12 µg/mouse), and pT2/shp53/mPDGF (0.12 µg/mouse), and injected into the right lateral ventricle of neonates, as described in Kosaka 2014.
Authentication	The cell lines were authenticated via RNA-seq and whole-exome sequencing.
Mycoplasma contamination	All lines tested negative for mycoplasma contamination.
Commonly misidentified lines<br>(See ICLAC register)	None.

Animals and other research organisms

Policy information about studies involving animals; ARRIVE guidelines recommended for reporting animal research, and Sex and Gender in Research

Laboratory animals	10 weeks-old C57BL/6J were housed at temperatures of 65-75°F (~18-23°C) with 40-60% humidity and a 14-hour light/10-hour dark cycle.
Wild animals	None
Reporting on sex	Only female mice were used in this study.
Field-collected samples	None
Ethics oversight	All animal experiments were conducted in compliance with protocols approved by the Institutional Animal Care and Use Committee (IACUC) at UCSF, following the National Institutes of Health Guidelines for animal care.

Note that full information on the approval of the study protocol must also be provided in the manuscript.
